# Aberrant claustrum structure in preterm-born neonates: an MRI study

**DOI:** 10.1016/j.nicl.2022.103286

**Published:** 2022-12-08

**Authors:** Antonia Neubauer, Aurore Menegaux, Jil Wendt, Hongwei Bran Li, Benita Schmitz-Koep, Tobias Ruzok, Melissa Thalhammer, David Schinz, Peter Bartmann, Dieter Wolke, Josef Priller, Claus Zimmer, Daniel Rueckert, Dennis M. Hedderich, Christian Sorg

**Affiliations:** a Department of Neuroradiology, Klinikum Rechts der Isar, Technical University of Munich, Germany; b School of Medicine, Klinikum Rechts der Isar, Technical University of Munich, Germany; c Department of Informatics, Technical University of Munich, Germany; d Department of Quantitative Biomedicine, University of Zurich, Switzerland; e Department of Neonatology and Pediatric Intensive Care, University Hospital Bonn, Germany; f Department of Psychology, University of Warwick, Coventry, UK; g Warwick Medical School, University of Warwick, Coventry, UK; h Department of Psychiatry and Psychotherapy, Klinikum Rechts der Isar, Technical University of Munich, Germany; i Neuropsychiatry, Charité – Universitätsmedizin Berlin and DZNE, Berlin, Germany; j University of Edinburgh and UK DRI, Edinburgh, UK; k Department of Computing, Imperial College London, UK

**Keywords:** Claustrum, Neonate, Brain development, Preterm birth, Magnetic resonance imaging, Subplate neurons, dHCP, developing Human Connectome Project, Draw-EM, developing brain region annotation with expectation–maximization, DWI, diffusion-weighted imaging, FA, fractional anisotropy, FT, full-term, GA, gestational age, GLM, general linear model, GM, gray matter, MD, mean diffusivity, MRI, magnetic resonance imaging, NODDI, neurite orientation dispersion and density imaging, pre-OL, pre-oligodendrocyte, PT, preterm, T2w, T2-weighted, TBV, total brain volume, TE, echo time, TR, repetition time

## Abstract

The human claustrum is a gray matter structure in the white matter between insula and striatum. Previous analysis found altered claustrum microstructure in very preterm-born adults associated with lower cognitive performance. As the claustrum development is related to hypoxia–ischemia sensitive transient cell populations being at-risk in premature birth, we hypothesized that claustrum structure is already altered in preterm-born neonates.

We studied anatomical and diffusion-weighted MRIs of 83 preterm- and 83 term-born neonates at term-equivalent age. Additionally, claustrum development was analyzed both in a spectrum of 377 term-born neonates and longitudinally in 53 preterm-born subjects. Data was provided by the *developing Human Connectome Project*.

Claustrum development showed increasing volume, increasing fractional anisotropy (FA), and decreasing mean diffusivity (MD) around term both across term- and preterm-born neonates. Relative to term-born ones, preterm-born neonates had (i) *increased* absolute and relative claustrum volumes, both indicating increased cellular and/or extracellular matter and being in contrast to other subcortical gray matter regions of decreased volumes such as thalamus; (ii) lower claustrum FA and higher claustrum MD, pointing at increased extracellular matrix and impaired axonal integrity; and (iii) aberrant covariance between claustrum FA and MD, respectively, and that of distributed gray matter regions, hinting at relatively altered claustrum microstructure.

Results together demonstrate specifically aberrant claustrum structure in preterm-born neonates, suggesting altered claustrum development in prematurity, potentially relevant for later cognitive performance.

## Highlights

• Claustrum development is related to subplate neurons and pre-oligodendrocytes.
• Both cell populations are at-risk in preterm birth.
• Anatomical and diffusion MRI mapped claustrum structure in preterm-born neonates.
• Claustrum macro-, micro-, and covariance structure were altered after preterm birth.
• Data suggest aberrant claustrum development in prematurity.

## 1 Introduction

Preterm birth – i.e., birth before 37 weeks of gestational age (GA) – is frequent with a worldwide prevalence of about 11 % (Chawanpaiboon et al., 2019). Beyond increased risks for multiple somatic impairments, preterm birth increases risks for impaired neurocognitive outcomes such as lower IQ and altered brain structure or development (Tauzin, 2015, Twilhaar et al., 2018, Volpe, 2019, Wolke et al., 2019). Several brain structures are affected in preterm-born individuals, observed not only during the neonatal period and in infancy but also in childhood, adolescence, and at young adult age (de Kieviet et al., 2012, Dimitrova et al., 2020, Hedderich et al., 2020, Nosarti et al., 2008, Schmitz-Koep et al., 2021a). Impacted regions are gray matter cortex, e.g., aberrant gyrification or thickness (Hedderich et al., 2020, Hedderich et al., 2019, Zhang et al., 2015), white matter tracts, e.g., altered microstructure and connectome (Ball et al., 2012, Kinney and Volpe, 2018, Menegaux et al., 2020, Meng et al., 2016), and gray matter subcortical regions, e.g., impaired thalamus, striatum, and cholinergic basal forebrain (Ball et al., 2012, Grothe et al., 2017, Schmitz-Koep et al., 2021b). Critically for the current study, we recently found aberrant claustrum microstructure in very preterm-born *adults*, with these changes being associated with preterm GA and cognitive performance in adulthood (Hedderich et al., 2021). As in the period of preterm birth, the claustrum development overlaps with transient cell populations, namely subplate neurons and pre-oligodendrocytes, particularly vulnerable to adverse events like hypoxia–ischemia and inflammation, which frequently co-occur with prematurity (Bruguier et al., 2020, McClendon et al., 2017, Puelles, 2014, Volpe, 2019), we suggested an early impairment of the claustrum in prematurity. The current study is testing this hypothesis.

In more detail, the claustrum is a tiny gray matter region between the extreme and external capsule adjacent to insula, and striatum (Puelles, 2014). Although ignored for a long time, the last two decades of increasing neuroscientific research on the claustrum revealed its highly important role in *basic forebrain functioning* (Crick and Koch, 2005). For example, the claustrum controls – via cortical inhibition – both basic attention processes during wakefulness, slow wave activity spreading during Non-REM sleep, and coordinated activity during REM sleep (Brown et al., 2017, Krimmel et al., 2019, Narikiyo et al., 2020, Renouard et al., 2015, Smith et al., 2020, White et al., 2018). Correspondingly, the claustrum is, relative to its volume, one of the *most connected regions* in the forebrain (Torgerson et al., 2015), with mostly bi-directional connections to not only primary and associative cortices but also the hippocampus and subcortical regions like the thalamus, amygdala, and neuromodulatory nuclei (Jackson et al., 2020, Torgerson et al., 2015). The development of highly connected regions, like the claustrum, is affected by the functioning of white matter connections depending on pre-oligodendrocytes (pre-OLs) (Back and Volpe, 2018, Kinney and Volpe, 2018, Volpe, 2019). Pre-OLs are transient precursor cells of white matter myelin-producing and fiber-ensheathing OLs, with main activity during GA 24–32, i.e., overlapping with preterm birth (Volpe, 2019); they are particularly vulnerable to hypoxic-ischemic events (Back, 2017, Back et al., 2005, Back and Volpe, 2018, McClendon et al., 2017, Volpe, 2019). Furthermore, the claustrum *development* is linked with that of subplate neurons, a key target of prematurity-related hypoxic-ischemic events (Volpe, 1996). Subplate neurons form a transient layer under the cortical plate with its peak around GA 17–37 and influence the maturation of thalamocortical connections, cortical layering, and corticocortical connections (Hoerder-Suabedissen and Molnár, 2015, Kanold and Luhmann, 2010, Kostovic and Rakic, 1990, Luhmann et al., 2018, Molnár et al., 2020). Critically, intimate relations between the subplate and the claustrum cells were reported regarding early development, global connections, and shared gene expression patterns, e.g. the orphan nuclear receptor *Nr4a2/Nurr1* and *Sulf1* (Arimatsu et al., 2003, Bruguier et al., 2020, Hoerder-Suabedissen and Molnár, 2015, Luhmann et al., 2018, Milardi et al., 2015, Torgerson et al., 2015, Watson and Puelles, 2017). Some claustral cells might tangentially migrate into the subplate as a sub-population of subplate neurons (Watson and Puelles, 2017). Several studies showed that subplate neuron maturation is especially vulnerable to hypoxic-ischemic events and is therefore at high risk in preterm birth (Kinney et al., 2012, McClendon et al., 2017, McQuillen and Ferriero, 2005, Volpe, 1996).

Combining the finding of altered claustrum microstructure in preterm-born *adults* (Hedderich et al., 2021) with the close relation to vulnerable transient cell populations during development, we hypothesized that the claustrum structure is already aberrant in preterm-born neonates. To test this hypothesis, we performed a cohort study of T2- and diffusion-weighted MRI scans of currently available 83 preterm- (PT) born neonates as well as comparable 83 full-term- (FT) born neonates with term-equivalent scan age of the *developing Human Connectome Project* (dHCP, https://www.developingconnectome.org) by comparing claustrum macrostructure, microstructure, and structural relationship with other brain regions. As this is the first analysis of the human neonatal claustrum, the study provides contextual background about the claustrum development around term: first, we evaluated the claustrum structure across the spectrum of 377 term-born neonates of the dHCP around term; second, preterm claustrum development was examined by a longitudinal evaluation of 53 preterm-born neonates of the dHCP who passed a follow-up scan in the range of GA 38 to 45. To perform all analyses of the claustrum, we used a previously published deep learning algorithm to segment the neonate claustrum (Neubauer et al., 2022), which was adapted from the corresponding adult claustrum segmentation tool (Li et al., 2021). Claustrum macrostructure was assessed with volume estimations in T2-weighted MRI scans. Claustrum microstructure was assessed using fractional anisotropy (FA) and mean diffusivity (MD) measures derived from diffusion-weighted images (DWI). Both, MD and FA of gray matter, reflect water diffusion along cell membranes, which has been used to characterize gray matter microstructure in normally developing tissue as well as tissue impaired by hypoxic-ischemic events (Batalle et al., 2019, Le Bihan, 2003, Lee et al., 2021, Lee et al., 2020). Claustrum structural relationship to other brain regions was assessed by structural covariance analysis, i.e., analysis of correlations between claustrum and cortical and subcortical gray and white matter regions regarding macro- and microstructural metrics, respectively.

## 2 Materials and methods

### 2.1 Data: Neonates and MRI acquisition, preprocessing, and outcomes, not specified for the claustrum

This study is based on preterm- and term-born neonatal brain MRI of the second data release provided by the *developing Human Connectome Project*, dHCP, in 2019 (https://www.developingconnectome.org/) (Table 1A). Recruitment and scanning took place at the Evelina Newborn Imaging Centre, St. Thomas’ Hospital in London, United Kingdom (Hughes et al., 2017). Written consent by the parents was obtained before scanning (Hughes et al., 2017) and the study was approved by the National Research Ethics Committee (Bastiani et al., 2019). The sessions were conducted in natural sleep except for six term-born subjects who were sedated with chloral hydrate as their scans were indicated (Edwards et al., 2022). Scans were released with additional information about sex, birth age, and scan age, comprising a range of GA 29.3 to 45.1 (https://github.com/BioMedIA/dHCP-release-notes). We evaluated the following samples: first, to provide context information about claustrum development, the available spectrum of term-born subjects, i.e., 377 neonates (Table 1B), and a longitudinal cohort of preterm-born neonates, i.e., 53 neonates who were scanned twice (Table 1C), were analyzed. Second, for our main study about the impact of preterm birth on claustrum structure, we compared preterm-born neonates with an equally sized sample of term-born subjects (Table 1D). To reach age-equivalent conditions between both groups, each preterm-born subject that was scanned in the scan age range of the term-born subjects, i.e., GA 37.4 to 44.9 weeks, was included in this analysis resulting in 83 preterm-born subjects. Iteratively, each of them was matched with one term-born subject with a minimal scan age gap yielding 83 controls.

**Table 1:** Imaging Datasets of dHCP – in general and for the different analyses.

Dataset/Analysis		Image sequence	Term-born scans (male)	Mean scan age (±SD) in GA (weeks)	Preterm-born scans (male)	Mean scan age (±SD) in GA (weeks)
A	dHCP (second data release)	T2	378 (2 0 5)	41.1 (±1.7)	180 (1 1 2)	37.8 (±3.7)
		DWI	327 (1 7 5)	40.9 (±1.7)	163 (1 0 2)	37.6 (±3.6)
B	Context claustrum development 1: Claustrum structure across term-born spectrum	T2	377 (2 0 5)	41.1 (±1.7)		
		DWI	326 (1 7 5)	40.9 (±1.7)		
C	Context claustrum development 2: Preterm claustrum development (longitudinal)	1st T2			53 (34)	34.3 (±1.9)
		2nd T2			53 (34)	41.2 (±1.5)
		1st DWI			45 (28)	34.5 (±1.7)
		2nd DWI			45 (28)	41.3 (±1.5)
D	Main study: Impact of preterm birth	T2	83 (48)	41.1 (±1.7)	83 (51)	41.1 (±1.7)
		DWI	72 (41)	40.9 (±1.7)	72 (45)	41.0 (±1.7)

Overview of the data of the second data release of the dHCP and the scans for each analysis in this study. For each task, we included a maximum of one scan per subject except for the longitudinal analysis “Context claustrum development 2” which comprises two scans per subject. dHCP = developing Human Connectome Project, DWI = diffusion-weighted imaging, GA = gestational age, SD = standard deviation.

We focused on structural and diffusion-weighted MRI acquired with a neonatal 32-channel head coil and dedicated scanning conditions with a Philips Achieva 3T (Hughes et al., 2017). *Structural MRI:* Due to immature brain appearance in neonates, the preferred structural image sequence in such a young population is T2-weighted MRI (Dubois et al., 2021). The scans were acquired with a Turbo Spin Echo sequence in axial and sagittal planes (repetition time (TR) = 12 s, echo time (TE) = 156 ms, SENSE factor (axial) = 2.11, SENSE factor (sagittal) = 2.58, overlapping resolution = 0.8*0.8*1.6 mm^3^, reconstructed resolution = 0.5*0.5*0.5 mm^3^) (Makropoulos et al., 2018). All scans passed a preprocessing pipeline with rigid motion correction (Cordero-Grande et al., 2018), bias correction, specialized reconstruction, and visual quality control (Kuklisova-Murgasova et al., 2012, Makropoulos et al., 2018). Further, the structural MRI underwent brain extraction with the Brain Extraction Tool (Smith, 2002) and it was automatically segmented into 87 regions by the dHCP using the Draw-EM (Developing brain Region Annotation With Expectation-Maximization) algorithm (Gousias et al., 2012, Makropoulos et al., 2018, Makropoulos et al., 2014). We used this co-registration to extract values from other regions for reasons of comparison and to determine the total brain volume (all intracranial structures including brainstem and excluding extracranial background, cerebrospinal fluid, and ventricles).

*Diffusion-weighted MRI:* Diffusion-weighted images (DWI) were acquired with a monopolar spin-echo echo-planar imaging Stejksal-Tanner sequence based on multi-shell High Angular Resolution Diffusion Imaging (TR = 3.8 s, TE = 90 ms, isotropic resolution of 1.5 mm) (Bastiani et al., 2019, Makropoulos et al., 2018). Diffusion data were preprocessed by correction for eddy current, motion, and susceptibility-induced distortions by the dHCP (Bastiani et al., 2019). Mean diffusivity, MD, and fractional anisotropy, FA, maps were reconstructed with FMRIB Software Library (FSL; version 6.0.4) and transformed to T2w space as the native high-resolution space of claustrum segmentation. Transformation was conducted with provided rigid-body transformation (Bastiani et al., 2019) based on Greve and Fischl (2009), and Jenkinson et al. (2002). The image registration passed through a quality check by the dHCP (Bastiani et al., 2019). DWI comprises fewer samples than structural MRI corresponding to the number of scans that passed the respective quality control by the dHCP (Bastiani et al., 2019).

### 2.2 Claustrum segmentation and macro-/microstructural claustrum outcomes

**Fig. 1:**
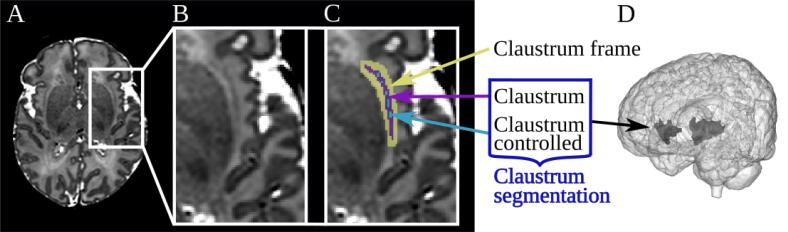
Claustrum segmentation in neonatal brain MRI. (A) T2-weighted axial MRI slice of a term-born neonatal brain at 38 weeks of gestational age (B) without and (C) with claustrum annotation. The *claustrum segmentation* applied in this study includes the label *claustrum* and *claustrum-controlled* (dark blue box). Both *claustrum frame* (yellow; the intersection of the insular white matter and an augmented claustrum segmentation of around two voxels on all sides minus the claustrum itself) and *claustrum-controlled* (light blue; claustrum voxels which were surrounded by other claustrum voxels for at least 90 %) were defined for control analyses. (D) A reconstructed 3D segmentation of the right and left claustrum. (For interpretation of the references to colour in this figure legend, the reader is referred to the web version of this article.)

To analyze the claustrum in newborn subjects, we applied three-dimensional segmentations in T2-weighted structural MR images (Fig. 1C, dark blue). Due to the intricate shape, the neonate claustrum is not listed in standard brain atlases or the Draw-EM coregistration (Makropoulos et al., 2018, Makropoulos et al., 2014). As manual segmentation both show substantial inter-rater variability and is very laborious, we used a previously published deep learning algorithm to segment the neonate claustrum (Neubauer et al., 2022) (Fig. 1C, dark blue). In more detail, using a deep learning segmentation approach, a convolutional neural network was pretrained with manual claustrum segmentations in adult scans and extensively evaluated; it performed clearly above manual segmentation including intra-rater reliability (Li et al., 2021). Then, using an additional transfer learning approach, the ‘adult-claustrum-focused’ network was adapted for neonate claustrum segmentation by training and optimizing with 20 scans of preterm-born neonates of the dHCP including adjusted hyperparameters and data augmentation methods (Neubauer et al., 2022). The resulting ‘neonate-claustrum-focused’ network was also extensively evaluated with 538 scans of the dHCP using different volumetric and spatial scores (Neubauer et al., 2022). Critically, automated neonate claustrum segmentation exceeded substantially the inter-rater reliability of manual segmentation (Neubauer et al., 2022). The code was made publicly available on GitHub (https://github.com/hongweilibran/claustrum_multi_view).

All segmentations passed strict visual quality control following a structured segmentation protocol with ITK-SNAP (version 3.6.0, www.itksnap.org) (Yushkevich et al., 2006) to ensure high accuracy in all scans (Neubauer et al., 2022). Additionally, to approximate the small shape of the claustrum and delimit the structure from surrounding white matter in microstructure analyses, two control segmentations were established (Fig. 1C, yellow and light blue): first, a white matter “claustrum frame” was defined as the intersection of the insular white matter and an augmented claustrum segmentation of around two voxels on all sides minus the claustrum itself; second, the “claustrum-controlled” segmentation was determined as claustrum voxels that were surrounded by other claustrum voxels for at least 90 %.

The claustrum segmentations were used for structure outcome definition, namely *claustrum macrostructure* (i.e., both claustrum absolute volume directly derived from the segmentation and claustrum relative volume that is derived from claustrum absolute volume divided by total brain volume (TBV)), and *claustrum microstructure* (i.e., MD and FA that are defined as the mean intensity in the labeled region in the corresponding diffusion map, respectively). Analyses were performed with the mean of right and left claustrum for an overview, and for right and left claustrum separately to identify possible differences.

### 2.3 Claustrum covariance analysis

The relationship of regional cortical and subcortical gray and white matter structure with claustrum structure was analyzed by structural covariance analysis (Berman et al., 2020). The Draw-EM coregistration was applied to each subject to parcellate the gray and white matter of the brain, excluding the segmented claustrum. Every region, except for cerebrospinal fluid and background areas, was correlated with the right and left claustrum, for the different macro- and microstructural measures of absolute volume, TBV-relative volume, MD, and FA, respectively. The calculated Pearson’s r correlation coefficients were transformed to z-scores via Fisher r-to-z-transformation.

### 2.4 Statistical analysis

Statistical analyses were performed with R (version 3.6.3). Thresholds for statistical significance were set to p < 0.05.

To provide *context information about claustrum development* around birth, we studied first, how claustrum *structure* depends on age around birth for a spectrum of term-born subjects. Therefore, we analyzed all term-born subjects with scans from GA 38 to 45 using a general linear model approach (GLM). Absolute claustrum volume, TBV-relative claustrum volume, MD, and FA were dependent variables, respectively. Scan age was the independent variable, and the model was corrected for sex and birth age. Second, concerning claustrum *development of preterm-born subjects* around birth, we examined longitudinal MRIs of the dHCP in preterm-born neonates. Paired t-tests were applied to compare first and second scans of preterm-born subjects scanned twice for a longitudinal study of the same variables, namely absolute claustrum volume, TBV-relative claustrum volume, MD, and FA. Subjects who were scanned twice were demographically compared with subjects scanned once to determine a possible bias. Complementary, all available scans of preterm-born neonates were included in linear mixed model analyses with the same metrics as mentioned above as dependent variables, respectively, and scan age as independent variable. The models were controlled for birth age, sex, and random intercepts for individual subjects.

For our *main analysis of the effect of preterm birth on neonate claustrum structure*, we tested for significant differences between preterm- and term-born neonates at term-equivalent scan age, to determine whether preterm birth influences relative or absolute claustrum volume, MD, and FA. We included all preterm-born newborns, who were scanned in the scan age range of term-born neonates (GA 37.4–44.9), and just as many term-born neonates with minimal scan age gap to gain term-equivalent conditions. A GLM approach was performed for absolute and TBV-relative claustrum volume, MD, and FA, respectively, controlled for sex and scan age.

*Control analyses:* To assess if the selection of control subjects had an impact on the results, the term-born neonates who were included as controls and the subjects who were not included were compared regarding their demographic characteristics. Furthermore, the GLM approach was repeated with all available term-born subjects as controls for the main analyses. Besides, to control the macrostructure analysis, the GLM analyses were conducted but instead of claustrum outcomes, values from other subcortical regions were selected as the dependent variable, namely TBV-relative thalamus and caudate nucleus volumes, to evaluate the specificity of claustrum volume changes. For microstructure analysis, the GLM analyses were repeated with claustrum-controlled segmentations (Fig. 1C, light blue), respectively, to minimize the effect of the surrounding. Furthermore, we specifically controlled for potential confounding effects of nearby regions’ microstructure. FA values of regions close to the claustrum were added as a further covariate in the GLM approach, namely the lentiform nucleus (i.e., putamen and globus pallidus), insular cortex, and white matter *claustrum frame* (Fig. 1C, yellow). All labels used above, except the claustrum, its frame, and controlled segmentation, were defined in the Draw-EM brain segmentation provided for each T2w scan by the dHCP (Makropoulos et al., 2018, Makropoulos et al., 2014). The Draw-EM classification does not include putamen alone, which has the largest border to the claustrum; therefore, we used the caudate nucleus or lentiform nucleus instead of the putamen, which both have been Draw-EM classified (Makropoulos et al., 2018, Makropoulos et al., 2014). For consistency between brain regions, we still refer to this atlas and provide exclusive claustrum frame correction for the directly surrounding area and, e.g., the caudate nucleus as a further control region.

Concerning *structural covariance analysis*, z-scores of regional correlation values with claustrum structure were tested for significant differences between preterm- and term-born neonates, with a significance threshold of p < 0.05. P-values of this analysis passed false discovery rate correction for multiple testing. The results were presented in brain plots made with ITK-SNAP (version 3.6.0, https://www.itksnap.org) (Yushkevich et al., 2006) and the Visualization Toolkit vtk (version 9.1.0) in python (version 3.8.10). The code is available on GitHub (https://github.com/cor2ni/3D_brain_plot).

## 3 Results

### 3.1 Context claustrum development 1: Claustrum structure in term-born neonates from GA 38 to 45

**Fig. 2:**
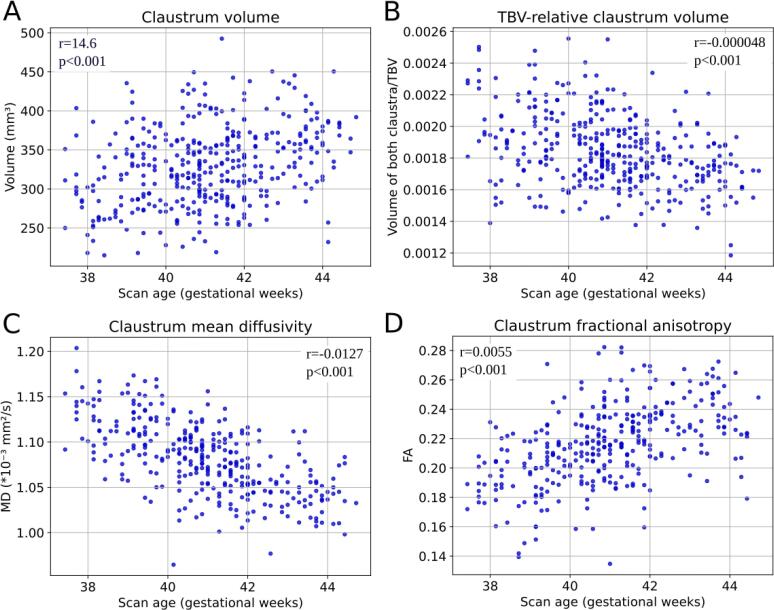
Claustrum structure around birth of term-born neonates. Macrostructure: (A) the absolute claustrum volume increases and (B) the total brain volume (TBV-) relative claustrum volume decreases in the early postnatal period in a spectrum of 377 term-born neonates. Microstructure: (C) the claustrum mean diffusivity (MD) decreases in the same period and (D) the claustrum fractional anisotropy (FA) increases with increasing scan age, shown in a spectrum of 326 term-born neonates. Regression coefficients r and p-values were calculated by a general linear model approach correcting for birth age and sex.

To set a contextual background for our study of the impact of prematurity on the claustrum in neonates, we first evaluated claustrum development by analyzing its structure in term-born neonates. The scans of term-born neonates were selected across the spectrum of gestational age (GA) from 38 to 45 weeks (i.e., 377 subjects). Claustrum macrostructure, i.e., the average absolute volume of both hemispheres, was determined using automated claustrum segmentation, and the results were plotted along scan age (Fig. 2A). The so defined distribution suggested an increase in claustrum volume with increasing age. To test this hypothesis, a GLM analysis was performed with mean claustrum volume as the dependent variable and scan age as an independent variable corrected for sex and birth age. The absolute claustrum volume significantly increased with increasing scan age (regression coefficient r = 14.6, p < 0.001; partial η^2^ = 0.11), indicating continuous volume increase of the claustrum around birth. Regarding right and left claustrum separately, both claustra showed a significant volume increase with older scan age with a superior growth rate in the right claustrum (r_right_ = 17.1, p_right_ < 0.001, r_left_ = 12.1, p_left_ < 0.001) (Fig. S1A).

To evaluate claustrum volume increase in relation to total brain volume, TBV, increase around birth (TBV is defined as the volume of the brain’s gray and white matter, including brainstem and excluding cerebrospinal fluid and ventricles) in term-born neonates, we used the same GLM approach as for absolute claustrum volume above but now with claustrum volume relative to TBV (i.e., the volume of right and left claustrum divided by TBV) as the dependent variable. The relative claustrum volume decreased significantly around term (r = –0.000048, p < 0.001; partial η^2^ = 0.15) (Fig. 2B). The relative claustrum volume also decreased significantly for right and left claustrum separately with a stronger decrease on the left side (r_right_ = -0.000018, p_right_ < 0.001, r_left_ = -0.000030, p_left_ < 0.001) (Fig. S1B). As the absolute claustrum volume was increasing, this suggests that claustrum volume increase is relatively inferior to TBV increase around term.

To assess changes in claustrum microstructure in term-born neonates around birth, we derived averaged claustrum mean diffusivity, MD, of DWI scans from term-born neonates (i.e., 326 subjects) across GA 38 to 45 and plotted these values (Fig. 2C). Again, to evaluate suggested claustrum MD decrease across the spectrum of age around birth, a GLM approach was applied. Mean claustrum MD (*10^-3^ mm^2^/s) was the dependent variable, scan age was the independent variable, and the model was corrected for sex and birth age. There was a significant negative association between scan age and averaged claustrum MD (r = –0.013, p < 0.001; partial η^2^ = 0.41), as well as right and left claustrum MD, respectively, (r_right_ = −0.012, p_right_ < 0.001, r_left_ = −0.014, p_left_ < 0.001) (Fig. S1C). These findings indicate decreasing claustrum MD in term-born neonates around birth. To evaluate the decreasing averaged claustrum MD in relation to general gray matter (GM) MD changes around birth, the GLM analysis was repeated with GM MD, i.e., averaged MD of cortical and subcortical GM, as a further independent variable in the GLM model. We found a similar negative relationship between scan age and claustrum MD (r = –0.0138, p < 0.001; partial η^2^ = 0.44), supporting the scan age-dependent decrease of claustrum MD in term-born neonates independent of GM MD changes.

To further study changes in claustrum microstructure in term-born neonates around birth, we derived averaged claustrum FA from DWI scans of the same subjects as for the MD analysis and plotted them across scan age (Fig. 2D). Then, to test for increasing claustrum FA around birth, a GLM analysis was conducted with mean claustrum FA as the dependent variable, scan age as the independent variable, and sex and birth age as covariates-of-no-interest. There was a significant positive relationship between scan age and averaged claustrum FA (r = 0.0055, p < 0.001; partial η^2^ = 0.26). This correlation was also seen for right and left claustrum FA separately with a slightly superior increase in the left claustrum (r_right_ = 0.0050, p_right_ < 0.001, r_left_ = 0.0060, p_left_ < 0.001) (Fig. S1D). These findings indicate increasing claustrum FA around birth. To evaluate the increasing averaged claustrum FA in relation to general GM FA changes around birth, we repeated the GLM analysis but added GM FA (i.e., averaged FA of cortical and subcortical GM) as a further independent variable in the GLM. Again, there was a positive relationship between scan age and claustrum FA (r = 0.0091, p < 0.001; partial η^2^ = 0.37), supporting the scan age-dependent increase of claustrum FA in term-born neonates independent of GM FA changes.

### 3.2 Context claustrum development 2: Development of claustrum structure in preterm-born neonates

**Fig. 3:**
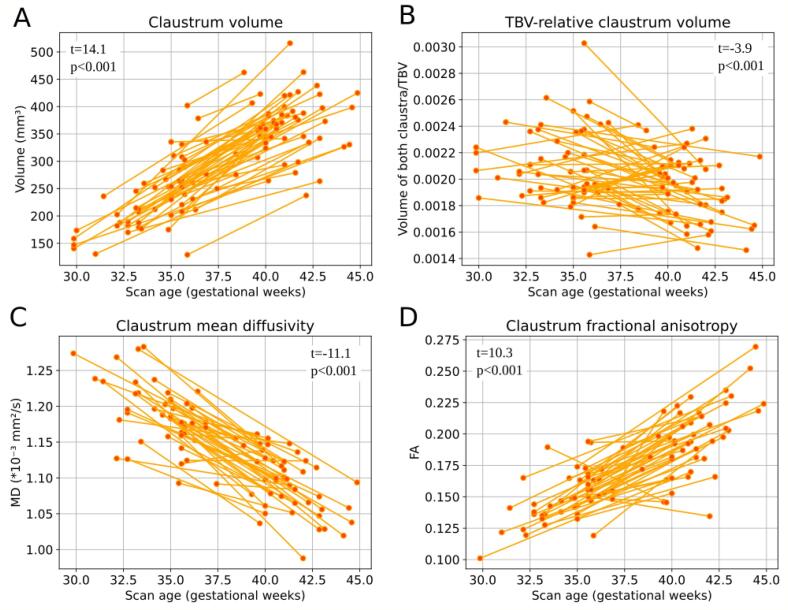
Claustrum development after birth of preterm-born neonates. Macrostructure: (A) Longitudinal examination of the claustrum structure development in preterm-born subjects. The absolute claustrum volume rose and (B) the total brain volume (TBV-) relative claustrum volume decreased in the postnatal period of 53 preterm-born subjects. Microstructure: (C) The claustrum mean diffusivity (MD) decreased in the first weeks after preterm birth, while (D) the mean claustrum fractional anisotropy (FA) rose, shown in 45 subjects. *T*-statistics and p-values were calculated by a paired *t*-test between the first and second scans, respectively.

As a second contextual background for our main study of prematurity impact on claustrum structure, we studied the development of claustrum structure around birth in *preterm-born* neonates. Therefore, scans at birth and a few weeks after birth were assessed, spanning a period from GA 30 to 45. To estimate the development of claustrum macrostructure, the averaged claustrum volume derived from two longitudinal T2-weighted scans was plotted (Fig. 3A), suggesting a consistent claustrum volume increase. To test this developing volume increase for significance, a paired *t*-test between the mean claustrum volume of the first and second scans of 53 preterm-born subjects scanned twice was performed. In terms of demographic characteristics of preterm-born neonates scanned twice or once, subjects who were scanned twice had a slightly lower average birth age and birth weight (Tables S1 and S2). There was a significant rise in absolute claustrum volume between the first and second scans (*t*-statistic = 14.1, p < 0.001). A linear mixed model approach including all available 128 preterm-born neonates, controlled for birth age, sex, and random intercepts for individual subjects, confirmed the claustrum volume increase with older scan age (regression coefficient r = 16.6, p < 0.001), indicating substantial postnatal claustrum growth after preterm birth. Larger claustrum volume with increasing scan age was also observed in right and left claustrum separately in paired t-tests (Fig. S2A) and linear mixed models (r_right_ = 17.8, p_right_ < 0.001, r_left_ = 15.1, p_left_ < 0.001) (Table S3), respectively, with a slightly superior growth rate in the right claustrum.

To evaluate claustrum volume development in relation to TBV development, the same paired *t*-test and linear mixed model approach was applied. Still, the absolute claustrum volume was replaced with relative claustrum volume (i.e., claustrum volume/TBV). We found that the relative claustrum volume significantly decreased around term (*t*-statistic = -3.9, p < 0.001) (Fig. 3B), indicating that claustrum volume increase from GA 30 to 45 is relatively smaller than the TBV increase in this period. This was supported by a negative regression coefficient in the linear mixed model (r = −0.000026, p < 0.001). Interestingly, this pattern seems valid for most neonates (Fig. 3B), but relative claustrum volume increased postnatally for 34 % of the preterm-born neonates scanned twice. The relative claustrum volume decrease was visible in right and left claustrum separately with a stronger effect in the left claustrum in the paired t-tests (Fig. S2B) and in the linear mixed models (r_right_ = −0.000008, p_right_ = 0.010, r_left_ = −0.000018, p_left_ < 0.001) (Table S3), respectively.

To estimate the development of claustrum microstructure after preterm birth, we focused on both, claustrum MD and FA, derived from DWI scans of preterm-born neonates, spanning the period of GA 30 to 45. Concerning MD, the claustrum MD course was plotted (Fig. 3C) and tested for significant MD decrease with a paired *t*-test for first and second scan of 45 subjects scanned twice as performed for claustrum volume increase. There was a significant reduction in claustrum MD (*t*-statistic = −11.1, p < 0.0001). Additionally, linear mixed models were conducted with all available scans of 119 preterm-born neonates correcting for birth age, sex, and random intercept for individual subjects. Again, there was a significant decrease in claustrum MD (r = −0.014, p < 0.001), indicating microstructural claustrum development after preterm birth. There was little difference between right and left claustrum MD in the paired t-tests (Fig. S2C) and linear mixed models (r_right_ = -0.014, p_right_ < 0.001, r_left_ = −0.015, p_left_ < 0.001) (Table S3). The same analysis was performed but with averaged claustrum FA instead of claustrum MD to further support such microstructural claustrum development. Fig. 3D suggests postnatal claustrum FA increase, which was confirmed by a paired *t*-test for its significance. There was a significant increase in claustrum FA (*t*-statistic = 10.3, p < 0.0001). This result was affirmed by the linear mixed model (r = 0.0067, p < 0.001), indicating postnatal claustrum microstructural development after preterm birth. The claustrum FA increase was slightly superior in the left claustrum than in the right regarding the paired *t*-test (Fig. S2D) and the linear mixed models (r_right_ = 0.0062, p_right_ < 0.001, r_left_ = 0.0071, p_left_ < 0.001) (Table S3).

### 3.3 Impact of preterm birth on claustrum structure

#### 3.3.1 Impact on macrostructure

**Fig. 4:**
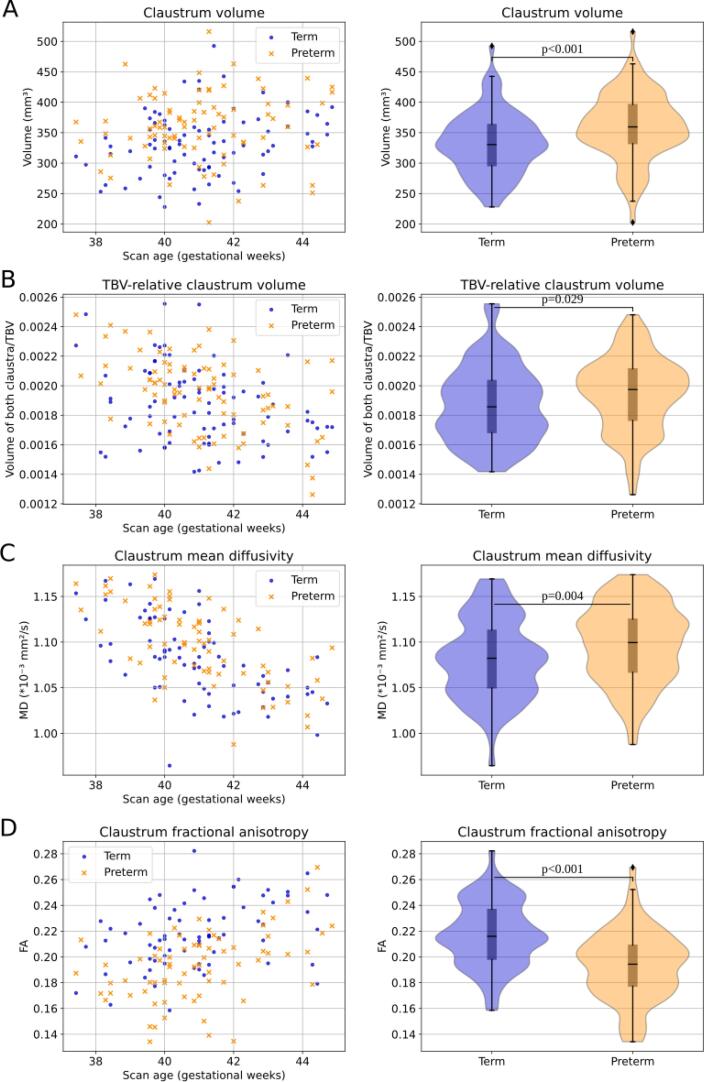
Impact of preterm birth on claustrum structure. We compared claustrum structure between groups of preterm- and term-born neonates. Significant differences (p < 0.05) between groups were tested with general linear models correcting for scan age and sex for each metric, respectively. Macrostructure: (A) The absolute claustrum volume and (B) the claustrum volume in relation to the total brain volume (TBV) were significantly increased after preterm birth tested in 83 preterm- and term-born neonates, respectively, at term-equivalent age. Microstructure: (C) The claustrum mean diffusivity (MD) was increased in preterm-born neonates while (D) the claustrum fractional anisotropy (FA) was decreased in preterm-born neonates tested in 72 preterm- and term-born neonates, respectively, at term-equivalent age.

After providing the context of both, term- and preterm-born neonate claustrum structure and development, we focused on the impact of preterm birth on the claustrum by comparing features of claustrum structure derived from term-equivalent scans of 83 preterm- (PT) and 83 term-born (full-term, FT) neonates, spanning GA 37.4 to 44.9. Birth age and birth weight of the 83 term-born neonates were comparable with the data of the other term-born subjects of the dHCP cohort making a selection bias less likely (Table S4). To examine the impact of preterm birth on claustrum macrostructure, first, averaged claustrum volumes were plotted (Fig. 4A). Then, we tested for volume differences across groups. GLM-based group comparison was performed with averaged claustrum volume as the dependent variable and preterm birth as the categorical independent variable, corrected for scan age and sex. There were significantly larger averaged claustrum volumes in preterm-born neonates (PT: 360 ± 55 mm^3^, FT: 332 ± 51 mm^3^; p < 0.001; partial η^2^ = 0.07). While the right claustrum volume was larger than the left claustrum in preterm- and term-born neonates, respectively, the preterm-born claustra were both larger than the term-born claustra, respectively, (p_right_ = 0.008, p_left_ < 0.001) (Table 2, Fig. S3A). This finding suggests larger claustrum volumes after preterm birth for term-equivalent comparison around birth.

**Table 2:** Impact of preterm birth on right and left claustrum structure separately.

Metric	Term (mean ± SD)	Preterm (mean ± SD)	p-value	partial η^2^
Right claustrum volume (mm^3^)	341 (±51)	363 (±58)	**0.008**	0.04
Left claustrum volume (mm^3^)	325 (±55)	357 (±56)	**<0.001**	0.08
TBV-relative right claustrum volume	0.00096 (±0.00013)	0.00098 (±0.00013)	0.183	0.01
TBV-relative left claustrum volume	0.00091 (±0.00015)	0.00097 (±0.00013)	**0.006**	0.05
Right claustrum MD (10^-3^ mm^2^/s)	1.08 (±0.04)	1.09 (±0.05)	**0.017**	0.04
Left claustrum MD (10^-3^ mm^2^/s)	1.09 (±0.05)	1.10 (±0.04)	**0.002**	0.07
Right claustrum FA	0.216 (±0.026)	0.187 (±0.028)	**<0.001**	0.26
Left claustrum FA	0.219 (±0.027)	0.199 (±0.029)	**<0.001**	0.13

Comparison of right and left claustrum structure between groups of 83 preterm- and 83 term-born neonates (72 preterm- and 72 term-born neonates for diffusion MRI). Significant differences (p < 0.05) between groups, written in bold, were tested with general linear models correcting for scan age and sex for each metric, respectively. FA = fractional anisotropy, MD = mean diffusivity, SD = standard deviation, TBV = total brain volume.

Although we carefully controlled for term-equivalence of comparisons and added scan age as a covariate as a further control, larger claustrum volumes in preterm-born neonates might be due to total brain changes. To control total brain changes, the same GLM approach as above was applied but absolute claustrum volume was replaced by TBV-relative claustrum volume (i.e., right and left claustrum volume/TBV; Fig. 4B). There were significantly larger relative claustrum volumes in preterm-born neonates (PT: 0.00195 ± 0.00025, FT: 0.00187 ± 0.00026; p = 0.029; partial η^2^ = 0.03), indicating that claustrum volumes are larger after preterm birth for term-equivalent comparison independent of TBV volumes. While the TBV-relative right claustrum volume (i.e., right claustrum volume/TBV; Fig. S3B) was not significantly different after preterm birth (p_right_ = 0.183), the TBV-relative left claustrum volume was significantly increased in preterm-born neonates (p_left_ = 0.006) (Table 2), suggesting a side specific effect.

When all available 377 term-born neonates were included as control subjects, both findings were reproducible, increased absolute claustrum volume (p < 0.001) and increased TBV-relative claustrum volume (p < 0.001) after preterm birth (Table S6), confirming the results in a larger cohort. Next, to control for the specificity of larger claustrum volume after preterm birth with respect to other subcortical regions, TBV-relative volumes of the thalamus and caudate nucleus were compared, respectively, between preterm- and term-born neonates by the same GLM analysis as for TBV-relative claustrum volume. We found significantly smaller relative thalamus volume (p = 0.001; partial η^2^ = 0.07) and significantly smaller relative caudate nucleus volume (p = 0.016; partial η^2^ = 0.04) in preterm-born neonates. Both results indicate the specificity of *larger* term-equivalent claustrum volume after preterm birth.

##### 3.3.1.1 Impact on microstructure

To examine prematurity impact on claustrum microstructure, we compared claustrum MD and FA, respectively, derived from term-equivalent scans of 72 preterm- and 72 term-born neonates, spanning the gestational age from 37.4 to 44.9 weeks. Birth age and birth weight of the 72 term-born neonates were comparable with the data of the other term-born subjects of the dHCP cohort making a selection bias less likely (Table S5). Concerning MD, we first plotted averaged claustrum MD (Fig. 4C) and tested then for MD differences across groups. A GLM analysis was performed with mean claustrum MD as the dependent variable and preterm birth (categorical) as the independent variable. Scan age and sex were included as covariates-of-no-interest. There was significantly larger mean claustrum MD in preterm-born neonates (PT: 1.10 ± 0.04 *10^-3^ mm^2^/s, FT: 1.08 ± 0.04 *10^-3^ mm^2^/s; p = 0.004; partial η^2^ = 0.06). Right and left claustrum MD separately showed little differences and were both significantly increased after preterm birth (p_right_ = 0.017, p_left_ = 0.002) (Table 2, Fig. S3C). The increased mean claustrum MD after preterm birth was also present comparing the 72 preterm-born neonates with all available 326 term-born neonates (p < 0.001) (Table S6), supporting the previous results. These findings suggest altered claustrum microstructure after preterm birth for term-equivalent comparison.

To test how the mean claustrum MD change was related to general GM MD change, we performed the same GLM analysis as just described but included GM MD (i.e., averaged MD of cortical and subcortical GM) as an additional covariate. We did not find a significant group difference for claustrum MD (p = 0.255), indicating that claustrum MD changes are dependent on general GM MD changes after preterm birth.

To approach the problem of limited diffusion scan resolution in the boundary region of the claustrum, a claustrum-controlled segmentation was established (i.e., claustrum voxels that are surrounded by other claustrum voxels for at least 90 %). We repeated the GLM analysis with claustrum-controlled MD as the dependent variable, and sex and scan age as independent variables. The claustrum-controlled MD was significantly increased in preterm-born neonates (p < 0.0001; partial η^2^ = 0.11) (Fig. S4). Adding the GM MD in this model as a further covariate, there was no significant difference for claustrum-controlled MD (p = 0.102). These findings support the above result, suggesting claustrum MD alteration depending on general GM MD changes after preterm birth.

Next, we studied the impact of prematurity on claustrum microstructure in terms of claustrum FA. The same GLM analysis as for claustrum MD was performed but with claustrum FA as the dependent variable (Fig. 4D). There was a significantly lower mean claustrum FA in preterm-born neonates (PT: 0.193 ± 0.027, FT: 0.218 ± 0.025; p < 0.0001; partial η^2^ = 0.21). Right and left claustrum FA separately were also significantly decreased after preterm birth (p_right_ < 0.001, p_left_ < 0.001) with a stronger effect in the right claustrum (Table 2, Fig. S3D). These findings indicate altered claustrum microstructure after preterm birth.

To test whether the mean claustrum FA change was comparable to general GM FA change, we performed the same GLM analysis as just described but included GM FA as an additional covariate. There was still a significantly lower claustrum FA (p = 0.007), indicating that lower claustrum FA does not depend on GM FA changes.

FA values are particularly influenced by fibers, i.e., connections of, to, and from a region. To control whether lower claustrum FA might be confounded by FA of nearby gray or white matter structures, several covariates were added next to scan age and sex in above mentioned GLM approach on claustrum FA, respectively: There was still a significantly lower claustrum FA in preterm-born neonates when correcting for insular cortex FA (p < 0.0001; partial η^2^ = 0.31), when correcting for lentiform nucleus (i.e., putamen and globus pallidus) FA (p < 0.0001; partial η^2^ = 0.31), and when correcting for white matter claustrum frame FA (p < 0.0001; partial η^2^ = 0.21).

For additional validation regarding the boundary region of the claustrum, the claustrum-controlled segmentation was applied instead of claustrum FA in a GLM correcting for sex and scan age. We found a significantly lower claustrum-controlled FA in preterm-born neonates (p < 0.0001; partial η^2^ = 0.17) (Fig. S4), supporting the finding with the whole claustrum FA. Adding the GM FA as a covariate in this GLM, there was no significant group difference (p = 0.069). These results confirm that claustrum FA is lower after premature birth, partly in the frame of general GM FA alterations.

### 3.4 Impact of prematurity on claustrum structure-related brain development

**Fig. 5:**
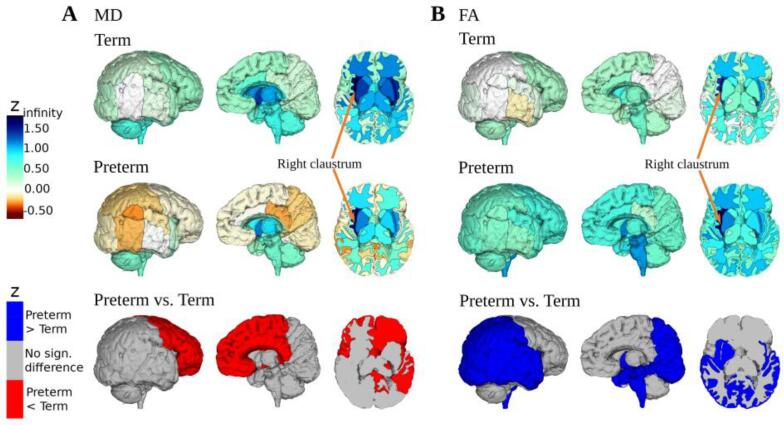
Impact of preterm birth on claustrum microstructure covariance. We plotted regional *right* claustrum microstructure covariance (for further structural measures and left claustrum: see Supplement). (A) Comparison of the covariance z-scores for mean diffusivity (MD) and (B) fractional anisotropy (FA) in preterm- and term-born neonates. Regions with significantly different z-scores were labeled in red or blue, respectively. (For interpretation of the references to colour in this figure legend, the reader is referred to the web version of this article.)

To analyze the impact of prematurity on the relationship of regional brain structure with claustrum structure, structural covariance analysis was performed for brain regions covering the whole brain. For each metric, i.e., absolute and TBV-relative volume, MD, and FA, respectively, each brain region was correlated with the corresponding measure of right and left claustrum across subjects of both groups, respectively (Table 1D, Table 3, and Fig. 5; brain plots for each metric for right and left claustrum can be found in the Supplement Figs. S5-8, all correlation coefficients and p-values are presented in Tables S7-10). The calculated Pearson’s r correlation coefficients were Fisher r-to-z-transformed and tested for significant differences between preterm- and term-born neonates for all regions, respectively, with subsequent false discovery rate correction because of multiple testing.

**Table 3:** Averaged regional structural covariance with the claustrum for cortical gray matter, subcortical gray matter, and white matter.

Group	Metric	Cortical GM	Subcortical GM	White matter
Preterm	Volume	0.44 (±0.10)	0.57 (±0.18)	0.49 (±0.14)
Term	Volume	0.43 (±0.10)	0.59 (±0.09)	0.43 (±0.16)
Preterm	Relative volume	−0.15 (±0.20)	0.12 (±0.25)	0.20 (±0.19)
Term	Relative volume	−0.17 (±0.14)	0.24 (±0.10)	0.09 (±0.16)
Preterm	MD	**0.08 (±0.29)^11/34^**	**0.70 (±0.25)^5/12^**	**0.68 (±0.21)^7/33^**
Term	MD	**0.40 (±0.23)**	**1.03 (±0.16)**	**0.99 (±0.19)**
Preterm	FA	**0.61 (±0.15)^25/34^**	**0.72 (±0.19)^4/12^**	**0.89 (±0.19)^3/33^**
Term	FA	**0.17 (±0.14)**	**0.44 (±0.23)**	**0.74 (±0.16)**

Structural covariance analysis between right claustrum structure and cortical gray matter (GM), subcortical GM (excluding the claustra themselves), and white matter structure in preterm- and term-born neonates. Correlation coefficients were calculated for absolute volume, total brain volume (TBV)-relative volume, mean diffusivity (MD), and fractional anisotropy (FA), respectively. The mean z-score (±standard deviation) is listed for each tissue group. Groups including subregions with significantly different z-scores after preterm birth are labeled in bold. The count of the affected subregions is denoted by a superscript number in the preterm rows: the first number refers to the count of significantly affected subregions, and the second number to the count of all subregions belonging to this group. The subregions refer to the listed regions in the Supplement (Table S7-10). E.g., regarding the row “Preterm MD” and the column “Cortical GM”, the cell content is “0.08 (±0.29)^11/34^”. “0.08 (±0.29)” is the average (±standard deviation) of the z-scores between the right claustrum and the 34 cortical GM subregions (listed in Table S7-10). The superscript numbers “11/34” specify how many subregions show significantly different z-scores in preterm-born neonates in comparison with term-born controls. Thus, we found significant covariance differences in eleven out of 34 cortical GM subregions after preterm birth. The cell content is written in bold because of these significant differences between preterm- and term-born neonates (in eleven subregions). There were no significant differences regarding the metrics volume and relative volume.

Regarding the absolute volume covariance, there was no significant difference between preterm- and term-born newborns for any brain region. All covariance z-scores, for cortical and subcortical GM, and WM, were positive with the highest mean (±standard deviation) correlation with subcortical GM (PT: 0.57 (±0.18), FT: 0.59 (±0.09)). Claustrum volume covariance with cortical GM was relatively uniform in preterm- and term-born subjects across the cortex (PT: 0.44 (±0.10), FT: 0.43 (±0.10)). These findings indicate a growing trend around term in the whole brain without a specific impact of preterm birth on the regional relationship with claustrum macrostructure.

Concerning the TBV-relative volume covariance, there was also no significant difference between preterm- and term-born neonates. We found negative and positive correlations in all tissue types, respectively. However, most cortical GM regions, especially the parietal cortex, showed negative z-scores and the mean coefficient was negative in preterm- and term-born subjects (PT: −0.15 (±0.20), FT: −0.17 (±0.14)). These findings indicate that cortical regions and claustrum show reverse TBV-relative volume trajectories around term without a significant impact by preterm birth.

Regarding MD covariance, the correlation of the claustrum MD with MD of the frontal, partly parietal, and temporal lobe, and cingulate gyrus, gray and white matter, subcortical GM like the amygdala, caudate nucleus, and subthalamic nucleus was significantly decreased after preterm birth. There were more significant differences for the right claustrum (23 regions) than the left (11 regions) (Table S9). The claustra showed positive correlations with cortical GM in term-born neonates (FT: 0.40 (±0.23)), while the z-scores for preterm-born subjects were partly negative (PT: 0.08(±0.29)). There was a high positive correlation with the subcortical gray and white matter in both groups with higher values in the full-term group (Table 3). This finding suggests an altered regional relationship of microstructure with that of the claustrum in preterm-born neonates for distributed cortical and subcortical regions.

Concerning FA covariance, the correlation between the right or left claustrum and parts of the parietal, temporal, occipital, parahippocampal lobe, hippocampus, and insula gray and white matter as well as the amygdala, subthalamic nucleus, and lentiform nucleus were significantly increased after preterm birth. Again, there were more significant differences noted in the right claustrum (33 regions including the brainstem) than in the left (10 regions) (Table S10). While the claustra of preterm-born subjects were positively correlated with all cortical (PT: 0.61 (±0.15)) and subcortical GM and WM regions, the term-born subjects showed partly negative z-scores with cortical GM (FT: 0.17 (±0.14), min/max: −0.11/0.58). Again, this finding suggests an altered regional relationship of microstructure with that of the claustrum in preterm-born neonates for distributed cortical and subcortical regions.

## 4 Discussion

Using term-equivalent T2-weighted anatomical and diffusion-weighted MRI scans of preterm- and full-term born neonates, we describe for the first time altered claustrum structure in preterm-born neonates. In particular, claustrum volume was increased after preterm birth, while volumes of other subcortical structures such as the thalamus or caudate nucleus were decreased. Furthermore, claustrum FA was decreased, and MD was increased after prematurity. While larger claustrum volumes indicate an increase of cellular and/or extracellular matter, lower fractional anisotropy and higher mean diffusivity suggest increased extracellular matrix and impaired axonal integrity. Performing a covariance analysis of the claustra with cortical and subcortical gray and white matter, aberrant microstructural covariance in cortical and subcortical regions hints at altered claustrum microstructure relative to that of widespread gray matter (GM). Taken together, these results indicate early alteration of the claustrum structure in prematurity. Data suggest impaired claustrum development around birth, speculatively due to overlapping vulnerability of both, subplate neuron and pre-oligodendrocyte pathways, which are critical for claustrum development. Furthermore, on the functional level, these results may indicate clinical implications for claustrum functions, namely attention and slow wave sleep processes.

### 4.1 Altered claustrum macrostructure in preterm-born neonates

Using T2-weighted neonatal brain MRI, we found absolute and TBV-relative *higher* claustrum volumes in preterm-born neonates compared with term-born ones. The observed differences were dominated by the left claustrum. However, the absolute right claustrum volume was also significantly increased after preterm birth. The volume differences between the right and left claustrum are in line with findings in adults in that the deviations vary between studies (Berman et al., 2020, Milardi et al., 2015, Torgerson et al., 2015). Our result is not influenced by scan age differences, as we carefully controlled for the term equivalence of scans. It is also not influenced by sex, for which we statistically controlled. The finding contrasts with volumes of other subcortical (neighboring) GM regions, which have – as expected and supported by other studies – *lower* volumes in preterm-born neonates. For example, TBV-relative volumes were reduced in the caudate nucleus and thalamus in the considered preterm-born neonates.

The finding of an increased deep GM volume in preterm-born neonates around term is contrary to previously reported trends. For example and in line with our results, Ball et al. (2012) found decreased thalamus volume in preterm-born neonates around term. Other studies reported generally decreased deep GM volume in preterm-born neonates compared to term-equivalent term-born subjects (Inder et al., 2005, Makropoulos et al., 2016). Asking for increased volumes in preterm-born neonates in particular, several studies reported increased volumes of cerebrospinal fluid but not GM structures (Inder et al., 2005, Makropoulos et al., 2016) except for one study which detected increased regions related to visual processing (Padilla et al., 2015). Expanding the observation group to preterm-born children and adults, global volume examinations showed exclusively increased volumes for selected cortical structures such as temporal and frontal lobe GM and cingulate gyrus (Meng et al., 2016, Nosarti et al., 2008).

Regarding the claustrum volume in adults, Hedderich et al. (2021) reported comparable volumes between preterm- and term-born subjects, suggesting that the effect of increased volume in preterm-born neonates is temporary. It is unclear how claustrum volumes develop during infancy, childhood, and adolescence.

Taken together, there is a specifically increased MRI-based volume signal of the claustrum after preterm birth. The observed claustrum volume increase could be attributed to the acute or subacute reactions of cellular and extracellular components to events of preterm birth. To differentiate these parts, we also performed a microstructural analysis.

### 4.2 Altered claustrum microstructure in preterm-born neonates

Analyzing neonatal diffusion MRI, we demonstrated altered claustrum microstructure, namely decreased claustrum FA and increased MD in preterm-born neonates compared to term-born neonates. While the claustrum MD deviation after preterm birth was slightly larger for the left claustrum, the claustrum FA deviation was dominated by the right claustrum. Our analysis was statistically controlled for sex, and scan age by carefully choosing term-equivalent scans. Furthermore, control analyses with the claustrum frame and the inner part of the claustrum, named claustrum-controlled, reduced the effect of nearby gray and white matter regions’ microstructure, especially corticostriatal fibers in the external capsule (Kostović et al., 2022, Schmahmann and Pandya, 2006), and corticocortical connections, e.g., frontotemporal fibers, in the extreme capsule (Mars et al., 2016, Petrides and Pandya, 2007). Although absolute results with these control segmentations partly deviated, the main finding of altered claustrum microstructure after preterm birth could be confirmed. As fiber tracts pervading the claustrum (Petrides and Pandya, 1988, Schmahmann and Pandya, 2006) cannot be fully excluded, the potential impact of axons on MD and FA values is also discussed below.

Decreased claustrum FA after preterm birth is consistent with previous studies dealing with deep GM structures of preterm-born neonates, which showed lower FA in the basal ganglia and thalamus in the first week of human neonates suffering hypoxic-ischemic events (Ward et al., 2006) and in the striatum of a hypoxic-ischemic animal model (Lee et al., 2021). In contrast, the claustrum FA in preterm-born adults was comparable with term-born controls (Hedderich et al., 2021), suggesting a transient microstructural reaction in the neonatal claustrum.

In addition, claustrum MD was absolutely increased. This increase was comparable with increased MD of other GM regions in preterm neonates as there was no significant difference after controlling for cortical and deep GM MD. Further studies investigating MD of deep GM structures in preterm-born neonates found increased thalamus MD around term (Ball et al., 2012, Rose et al., 2014) and more differentiated, equal, or decreased MD in basal ganglia and thalamus in the first week after hypoxic-ischemic brain injury but increased MD in these regions after two or three weeks (Rutherford et al., 2004, Ward et al., 2006), matching our results in preterm-born neonates around term. Our study is in line with the absolutely increased claustrum MD after preterm birth in adults (Hedderich et al., 2021), suggesting a long-term effect.

We interpreted FA and MD shifts as microstructural alterations on a cellular level. Both metrics represent the diffusion of free water molecules restricted by membranes of cell somata, dendrites, and axons with and without myelin (Beaulieu, 2002, Le Bihan, 2003). Higher MD is related to broader omnidirectional movement, typically interpreted as lower cellularity (Ball et al., 2012, Batalle et al., 2019, Hedderich et al., 2021). It can also reflect less myelination and more tissue water (Beaulieu, 2002, Dimitrova et al., 2020). In contrast, FA is a metric for how directional the water movement is (Beaulieu, 2002). It is mainly influenced by the coherence of axons and their myelination (Beaulieu, 2002), and neurite density and orientation dispersion (Zhang et al., 2012). A possible explanation for decreased FA in GM is lower axonal integrity of local or pervading neurons (Beaulieu, 2002, Eaton-Rosen et al., 2015, Ward et al., 2006, Zhang et al., 2012). A study in piglets suffering hypoxic-ischemic events showed decreased FA in the caudate nucleus and putamen correlated with swollen astrocytes, myelin impairment, and loss of oligodendrocytes (Lee et al., 2021). However, the latter study examined the brains within four days after an adverse event and the suggested underlying moderate cytotoxic edema did not show a significant effect on MD contradicting our finding of increased MD (Lee et al., 2021). Given these examples, decreased claustrum FA and increased MD in neonatal deep GM after preterm birth might be attributed to microstructural disturbances, including lower cell density and impaired connectivity in the claustrum. Another explanation could be a developmental delay of the claustrum after preterm birth based on the background analysis showing FA increase and MD decrease over time. However, this explanation disagrees with the macrostructural finding.

### 4.3 Altered microstructural covariance with the claustrum in preterm-born neonates

Correlating claustrum macro- and microstructure with other brain regions, the microstructural relations were impaired after preterm birth. While the MD covariance of spread cortical and subcortical regions was significantly decreased in preterm-born neonates, the FA correlation was partly increased. To exclude the effect of age, we chose age-equivalent scans for preterm- and term-born neonates. The multiple testing was statistically controlled by false discovery rate correction. In accordance with our results, altered covariance is common in developmental disorders (Alexander-Bloch et al., 2013). However, we could not find altered macrostructural covariance in preterm-born neonates as described for very preterm adolescents (Nosarti et al., 2011), possibly because of the younger subjects in our study.

We propose two non-exclusive interpretations of the altered microstructural covariance. First, as the subjects in this analysis were scanned in a range of more than seven weeks, there was a large variability within the groups due to ongoing brain development. In this context, high correlations could be interpreted as a parallel development of two regions (Alexander-Bloch et al., 2013). Correlation shifts in the preterm group suggest that preterm birth has a region-dependent impact on microstructural maturation (Alexander-Bloch et al., 2013, Nosarti et al., 2011). Alterations might arise directly from pathophysiological pathways of hypoxic-ischemic events in each region, in our case at least in the claustrum due to the regional findings reported above (Millar et al., 2017). Thereby, the vulnerability of a region depends on the genetically defined tissue properties like the expression of glutamate receptors (Back and Volpe, 2018, Millar et al., 2017). Together with the time point of damage, these conditions might result in altered development varying across the regions. This means in our case that claustrum development in preterm-born neonates might be distinctively impaired.

Second, the structural covariance of two regions could appear in the frame of connection-based networks (Alexander-Bloch et al., 2013, Berman et al., 2020, Mechelli et al., 2005). The claustrum and connected regions could be altered indirectly as a consequence of aberrant transient cell populations like pre-oligodendrocytes (pre-OLs) (Back and Volpe, 2018, Kinney and Volpe, 2018, Nosarti et al., 2011, Volpe, 2019) and subplate neurons (Bruguier et al., 2020, Kinney et al., 2012, Volpe, 1996). Damaged pre-OLs can lead to impaired connectivity and aberrant up- and downstream regions of affected connections (Kinney and Volpe, 2018). Impaired subplate neurons can result in altered cortical structure (Hoerder-Suabedissen and Molnár, 2015) and are also related to the claustrum (Bruguier et al., 2020). In this sense, the aberrant microstructural covariance after preterm birth could indicate altered connectivity, e.g., of the claustrum with spread cortical regions possibly related to functional interference.

Consistently for all brain regions significantly impacted by preterm birth, the MD covariance was decreased in preterm-born subjects while the FA covariance was increased. A possible explanation could be that claustrum MD is decreasing and claustrum FA is increasing around term, while cortical MD is also decreasing and cortical FA is not rising but decreasing up to GA 38 and stagnating or minimally rising afterward (Ball et al., 2013). Thus, when the brain is altered by preterm birth, the claustrum MD development diverges from cortical maturation, while the claustrum FA development converges to the cortical trend.

Furthermore, the significant MD differences were more pronounced in the frontal cortex, whereas the FA differences were present in the parietal and temporal regions, suggesting a difference between these areas. Dimitrova et al. (2021) described that the FA of parietal and temporal cortical areas shows a negative correlation with gestational age, and the MD of some frontal areas shows a negative correlation with gestational age but not vice versa. However, this difference was not visible in Ball et al. (2013). The effect was explained by different functions and extensive synaptogenesis specifically in sensory areas, e.g., because of extrauterine stimuli (Dimitrova et al., 2021, Huttenlocher and Dabholkar, 1997). The impact of preterm birth on the cortical microstructure is under debate: Ball et al. (2013) found increased MD and FA for all cortical regions while Bouyssi-Kobar et al. (2018) found increased MD in most regions but increased FA only in the primary motor cortex. A possible explanation for the variability of vulnerability across cortical regions may be the heterogenous maturation of the cortex (Dimitrova et al., 2021). The observation of the MD correlation difference in the frontal cortex in contrast to the FA correlation difference in parietal, temporal, and occipital areas might be explained by specific variations in the trajectories of radial organization of the cortex (McKinstry et al., 2002).

Additionally, we found more significant alterations correlating with the right claustrum than the left, suggesting a structural difference between the right and left hemispheres. Indeed, there is evidence of a volume asymmetry in the neonatal brain, with the left hemisphere being larger than the right (Gilmore et al., 2007), presumably due to genetic influences (Sun et al., 2005). The finding suggests that the right claustrum might be more damaged than the left.

Thus, preterm birth might impair the coordination of the development and connectivity between the claustrum and other brain regions. The exact mechanism behind the region-dependent alterations needs to be elucidated in further studies analyzing different brain networks in preterm- and term-born subjects.

### 4.4 Linking altered claustrum structure and structural relationship to gray matter regions – some speculations

Combining the observed macro- and microstructural claustrum alteration, and aberrant microstructural covariance, there are several imaginable but speculative pathophysiological explanations. We speculate to demonstrate how fruitful a multi-modal structural approach to the claustrum is both to generate new hypotheses and to inspire further microstructural investigations. This involves histological assessment including local cell-specific -omics analysis of preterm neonate claustrum and advanced diffusion MRI. The latter refers, e.g., to constrained spherical deconvolution and to neurite orientation dispersion and density imaging (NODDI) which can provide additional details about intra- and extra-neurite volume fraction (Zhang et al., 2012). Recent studies about prematurity applied this method to examine white and gray matter microstructure (Bastiani et al., 2019, Eaton-Rosen et al., 2015, Kelly et al., 2022, Kline et al., 2022, Pecheva et al., 2018, Wang et al., 2022). Thereby, NODDI metrics can be used complementary to the MD and FA metrics (Vaher et al., 2022).

To start with the comprehensive interpretation of the claustrum values collected so far, MD increase has been linked to lower cellularity (Ball et al., 2012, Batalle et al., 2019, Hedderich et al., 2021). This can be achieved by fewer cell somata and neurites and/or by a higher amount of extracellular matrix. Only cell loss is insufficient to explain the macrostructural volume increase. Instead, fluid accumulation conforms to increased absolute claustrum volume. Such larger distances of membranes could distract the usual directionality of water movement between neurites leading to lower FA in line with our results.

An inflammatory reaction may have caused an accumulation of the extracellular matrix. Secondary neuroinflammatory processes typically emerge as a response to impaired pre-OL development in prematurity induced by hypoxic-ischemic events (Volpe, 2019). Furthermore, affected pre-OLs result in reduced myelination (Volpe, 2019) of claustrum connections consistent with increased MD and decreased FA (Beaulieu, 2002, Dimitrova et al., 2020), although we should keep in mind that myelination is generally immature around term (Volpe, 2019). Neuroinflammatory processes could also be induced by hypoxic-ischemic effects on specific claustral cells, including subplate-derived cells, which are – like pre-OLs – particularly vulnerable to hypoxia–ischemia (Kinney et al., 2012, McClendon et al., 2017, McQuillen and Ferriero, 2005, Volpe, 2019).

However, we found a volume decrease in the likewise highly connected thalamus. One difference between the two structures is the cellular composition, e.g., the direct subplate neuron involvement in the immature claustrum (Bruguier et al., 2020, Puelles, 2014). It is speculation that this point might be critical. In other words, the specific overlap of both highly vulnerable pathways – pre-OLs and subplate neurons – in the claustrum might underpin the vulnerability of the claustrum.

### 4.5 Potential clinical implications of altered claustrum structure for developing claustrum function

The claustrum is suggested to play a role in both attention modulation during wakefulness and slow wave generation during Non-REM sleep (Brown et al., 2017, Liu et al., 2019, Narikiyo et al., 2020, Smith et al., 2020, White et al., 2018). These functions are fundamental for normal mental development and cognitive performance, which are at risk after preterm birth (Twilhaar et al., 2018, Wolke et al., 2019). Supporting the connection between claustrum and attention, Hedderich et al. (2021) showed a correlation between claustrum microstructure alteration and general cognitive performance, namely intelligence quotient, in very preterm-born adults. In the current study, claustrum macro- and microstructure was already aberrant in preterm-born neonates. Claustrum neurons emerge relatively early during brain maturation and develop spread cortical and subcortical connections (Bruguier et al., 2020, Jackson et al., 2020). We hypothesize that structural claustrum alterations in preterm-born neonates might predict the developing attention outcomes, based on these properties, of the first years after birth. To test this hypothesis, the release of data on attention and cognitive assessment 1.5 years after birth in the dHCP study is welcome.

Additionally, the duration of slow wave sleep after preterm birth might be related to claustrum alteration. A previous study found delayed slow wave sleep development in aggrieved neonates (Watanabe et al., 1974). Both, increased slow wave activity (Chan et al., 2020, Wehrle et al., 2017) and decreased slow wave duration (Perkinson-Gloor et al., 2015), were described in preterm-born children and adolescents. Based on these properties, further examination of sleep alteration after preterm birth is needed. We suggest that structural claustrum alterations in preterm-born neonates might contribute to slow wave sleep impairments after preterm birth.

Further work is required to test the viability of the claustrum as a prognostic marker of prematurity, possibly together with sleep assessment. One supporting point of these ideas is the specific volume increase of the claustrum in contrast to other GM subcortical regions.

### 4.6 Strengths and limitations

This study is based on a latest, public, large-scale dataset with a high image resolution and visual quality control (dHCP). However, some limitations should be taken into consideration. First, the claustrum is a delicate structure, so the segmentation is partly subjective despite the high image resolution. We performed automated segmentation to lower the impact of subjectivity in segmentation. Second, partial volume effects lead to a mixture of gray and white matter and might confound results. This might be a pronounced problem for diffusion space co-registration with limited resolution. To approach this issue, we controlled for surrounding gray and white matter in all analyses and applied a claustrum-controlled segmentation to further reduce the effect of boundary regions in microstructural evaluation. Third, the analysis of GM microstructure in diffusion-weighted images is confined by the applied metrics. Particularly, the FA values are relatively low (between 0.10 and 0.28), and interpretation is ambiguous in this range, at least in the white matter below 0.2 (Smith et al., 2006). Additionally, the reproducibility of FA in gray matter is restricted (Vollmar et al., 2010). Metrics like neurite orientation dispersion and density imaging in future studies of GM microstructure could improve the validity. Histological evaluation is needed for verification. Fourth, the statistical analyses with general linear models could be criticized for the inappropriate simplification of the data as solely linear functions. However, despite the large cohort, the risk of overfitting the scattered dataset with a more complex function is present. Thus, we decided to choose linear models for evaluation. Fifth, related to the previous point, the preterm- and control groups were scanned in a time range of several weeks. In this period, the claustrum structure changes and it is imaginable that the differences between both groups also change over time (Barkovich et al., 2006). As this would reduce the number of subjects for each analysis, we confined our test to the whole-time interval and plotted the data for a visual overview.

## 5 Conclusion

We demonstrated increased claustrum volume, aberrant claustrum microstructure, and altered microstructural covariance with the claustrum in preterm-born neonates in comparison with age-equivalent term-born controls. These findings indicate specifically altered claustrum structure in preterm-born neonates, suggesting an impaired claustrum development that potentially reflects the overlapping vulnerability of both subplate neuron and pre-oligodendrocyte pathways in the claustrum.

## 6 Data Availability Statement

All MRI data used in this study is part of a public data set of the dHCP. Feel free to register here for data access: https://www.developingconnectome.org/data-release/second-data-release/information-registration-and-download/.

The code for automated claustrum segmentation in neonatal brain MRI is available on GitHub: https://github.com/hongweilibran/claustrum_multi_view.

The code for 3D brain plots is published on GitHub: https://github.com/cor2ni/3D_brain_plot.

## Funding

This work was supported by the Deutsche Forschungsgemeinschaft (SO 1336/1-1 to C.S.), German Federal Ministry of Education and Research (BMBF 01ER0801 to P.B. and D.W., BMBF 01ER0803 to C.S.), the RECAP preterm project, an EU Horizon 2020 study (supported by grant 733280; D.W. and P.B.), and the Kommission für Klinische Forschung, Technische Universität München (KKF 8765162 to C.S., KKF 8700000474 to D.M.H., and KKF 8700000620 to B.S.-K.). Hongwei Bran Li is supported by Forschungskredit (Grant NO. FK-21-125) from University of Zurich.

## CRediT authorship contribution statement

**Antonia Neubauer:** Conceptualization, Methodology, Software, Formal analysis, Investigation, Writing – original draft, Writing – review & editing. **Aurore Menegaux:** Methodology, Software, Investigation, Writing – review & editing. **Jil Wendt:** Methodology, Validation, Writing – review & editing. **Hongwei Bran Li:** Methodology, Software, Investigation, Writing – review & editing, Funding acquisition. **Benita Schmitz-Koep:** Investigation, Writing – review & editing, Funding acquisition. **Tobias Ruzok:** Investigation, Writing – review & editing. **Melissa Thalhammer:** Investigation, Writing – review & editing. **David Schinz:** Methodology, Investigation, Writing – review & editing. **Peter Bartmann:** Investigation, Writing – review & editing, Funding acquisition. **Dieter Wolke:** Investigation, Writing – review & editing, Funding acquisition. **Josef Priller:** Methodology, Investigation, Writing – review & editing. **Claus Zimmer:** Methodology, Investigation, Writing – review & editing. **Daniel Rueckert:** Methodology, Investigation, Writing – review & editing. **Dennis M. Hedderich:** Conceptualization, Methodology, Investigation, Writing – original draft, Writing – review & editing, Supervision, Funding acquisition. **Christian Sorg:** Conceptualization, Methodology, Investigation, Writing – original draft, Writing – review & editing, Supervision, Funding acquisition.

## Declaration of Competing Interest

The authors declare that they have no known competing financial interests or personal relationships that could have appeared to influence the work reported in this paper.

## Data Availability

The authors do not have permission to share data.
