# Supplementary material for: Aberrant claustrum structure in preterm-born neonates: an MRI study
Source: Neuroimage Clin. 2022 Dec 8;37:103286. doi: 10.1016/j.nicl.2022.103286 (PMC9755238; doi:10.1016/j.nicl.2022.103286)
Supplement: Supplementary data 1 [file mmc1.pdf]

Supplement for

'Aberrant claustrum structure in preterm-born neonates: an MRI study'

By Antonia Neubauer et al.

Content:

Supplement figures and tables:

Fig. S1	Right and left claustrum structure development in term-born neonates.	p. 2
Table S1	Demographic characteristics of preterm-born neonates: T2-weighted scans.	p. 3
Table S2	Demographic characteristics of preterm-born neonates: diffusion-weighted scans.	p. 3
Table S3	Claustrum development in preterm-born neonates – linear mixed models.	p. 3
Fig. S2	Right and left claustrum structure development in preterm-born neonates.	p. 5
Table S4	Demographic characteristics of term-born neonates: T2-weighted scans.	p. 6
Table S5	Demographic characteristics of term-born neonates: diffusion-weighted scans.	p. 6
Table S6	Comparison of the claustrum structure of preterm-born and all term-born neonates.	p. 7
Fig. S3	Impact of preterm birth on right and left claustrum structure.	p. 8
Fig. S4	Impact of preterm birth on claustrum-controlled microstructure	p. 9
Fig. S5	Absolute volume correlation analysis with the claustrum.	p. 10
Table S7	Absolute volume correlation analysis with the claustrum: correlation coefficients.	p. 11
Fig. S6	Total brain volume relative volume correlation analysis with the claustrum.	p. 13
Table S8	Total brain volume relative volume correlation analysis with the claustrum: correlation coefficients.	p. 14
Fig. S7	Mean diffusivity correlation analysis with the claustrum.	p. 17
Table S9	Mean diffusivity correlation analysis with the claustrum: correlation coefficients.	p. 18
Fig. S8	Fractional anisotropy correlation analysis with the claustrum.	p. 21
Table S10	Fractional anisotropy correlation analysis with the claustrum: correlation coefficients.	p. 22

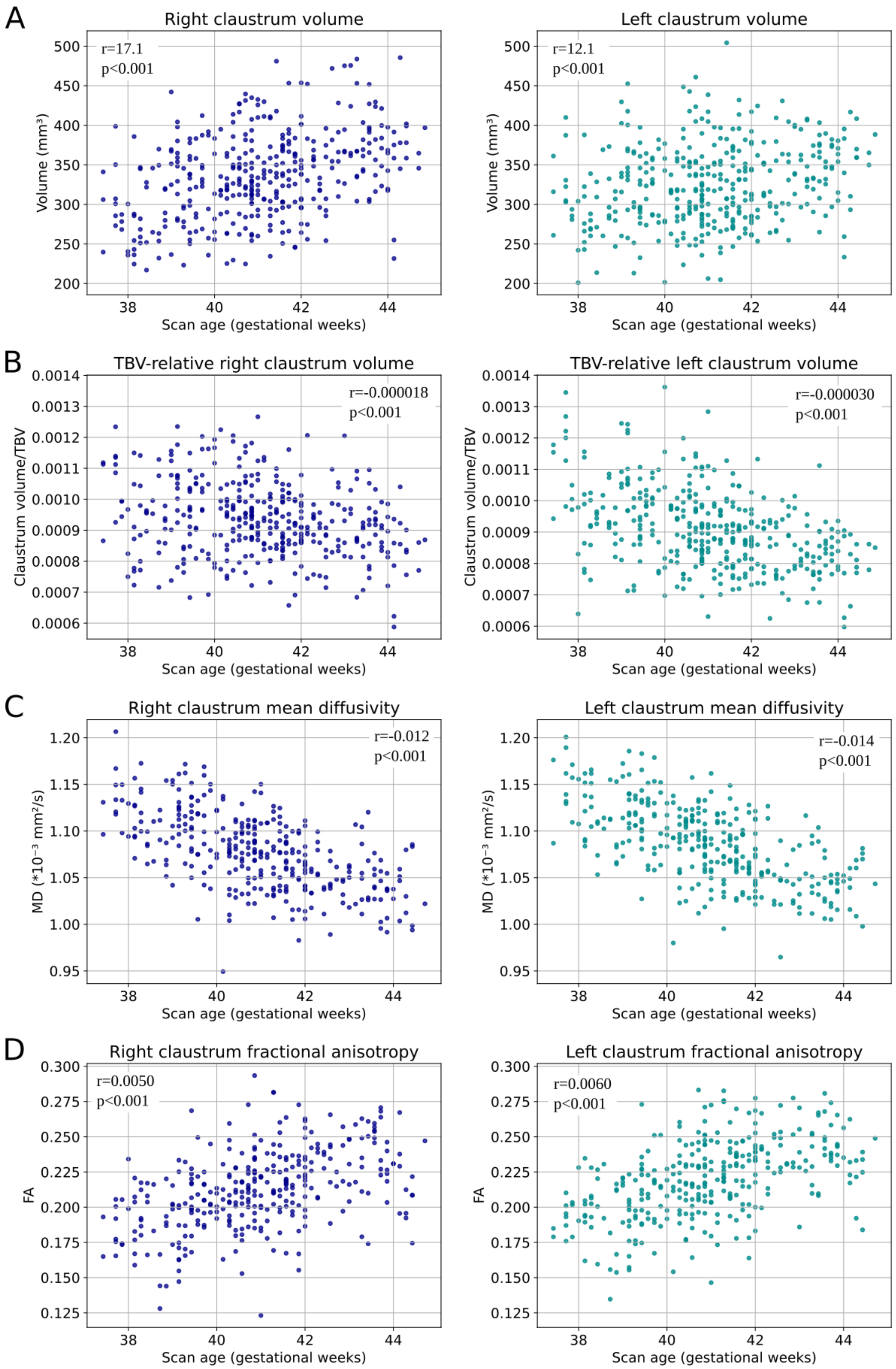
A
Right claustrum volume
r=17.1
p<0.001
Volume (mm³)
Scan age (gestational weeks)
Left claustrum volume
r=12.1
p<0.001
Volume (mm³)
Scan age (gestational weeks)
B
TBV-relative right claustrum volume
r=-0.000018
p<0.001
Claustrum volume/TBV
Scan age (gestational weeks)
TBV-relative left claustrum volume
r=-0.000030
p<0.001
Claustrum volume/TBV
Scan age (gestational weeks)
C
Right claustrum mean diffusivity
r=-0.012
p<0.001
MD (*10⁻³ mm²/s)
Scan age (gestational weeks)
Left claustrum mean diffusivity
r=-0.014
p<0.001
MD (*10⁻³ mm²/s)
Scan age (gestational weeks)
D
Right claustrum fractional anisotropy
r=0.0050
p<0.001
FA
Scan age (gestational weeks)
Left claustrum fractional anisotropy
r=0.0060
p<0.001
FA
Scan age (gestational weeks)

Fig. S1: Claustrum structure around birth of term-born neonates for right and left claustrum separately. Macrostructure: (A) the absolute claustrum volume increases and (B) the total brain volume (TBV-) relative claustrum volume decreases in the early postnatal period in a spectrum of 377 term-born neonates. Microstructure: (C) the claustrum mean diffusivity (MD) decreases in the same period and (D) the claustrum fractional anisotropy (FA) increases with increasing scan age, shown in a spectrum of 326 term-born neonates. Regression coefficients r and p-values were calculated by a general linear model approach correcting for birth age and sex.

Table S1: Demographic characteristics of preterm-born neonates of the developing Human Connectome Project, second data release, with T2-weighted scans. SD=standard deviation.

Preterm-born subjects	Subjects (Male)	Singleton status (Singles/Multiples)	Birth age (Mean ± SD)	Birth weight (kg)
Only one scan available	76 (44)	45 / 31	32.9 (±3.6)	1.95 (±0.72)
Two scans available	53 (34)	34 / 19	32.0 (±3.0)	1.69 (±0.66)

Table S2: Demographic characteristics of preterm-born neonates of the developing Human Connectome Project, second data release, with diffusion-weighted scans. SD=standard deviation.

Preterm-born subjects	Subjects (Male)	Singleton status (Singles/Multiples)	Birth age (Mean ± SD)	Birth weight (kg)
Only one scan available	75 (46)	46 / 29	32.9 (±3.5)	1.92 (±0.72)
Two scans available	45 (28)	29 / 16	32.2 (±3.0)	1.74 (±0.66)

Table S3: Claustrum development in preterm-born neonates. The claustrum development was estimated with linear mixed models to include all available scans of preterm-born neonates of the developing Human Connectome Project, second data release. That comprises 180 T2-weighted scans of 128 neonates and 163 diffusion-weighted scans of 119 neonates. In the

linear mixed models, the metrics absolute and relative claustrum volume, mean diffusivity (MD), and fractional anisotropy (FA) of right and left claustrum separately and averaged were the dependent variables, respectively. The scan age was the independent variable. The models were controlled for birth age and sex and expanded by random intercepts for individual subjects. The analysis was repeated for the mean claustrum metrics (r_{mean}, p_{mean}), right (r_{right}, p_{right}), and left claustrum (r_{left}, p_{left}), respectively. p=p-value, r=regression coefficient, TBV=total brain volume.

Metric	r_{mean}	p_{mean}	r_{right}	p_{right}	r_{left}	p_{left}
Claustrum volume (mm^3)	16.6	<0.001	17.8	<0.001	15.1	<0.001
TBV-relative claustrum volume	-0.000026	<0.001	-0.000008	0.010	-0.000018	<0.001
Claustrum MD (10^{-3} mm^2/s)	-0.014	<0.001	-0.014	<0.001	-0.015	<0.001
Claustrum FA	0.0067	<0.001	0.0062	<0.001	0.0071	<0.001

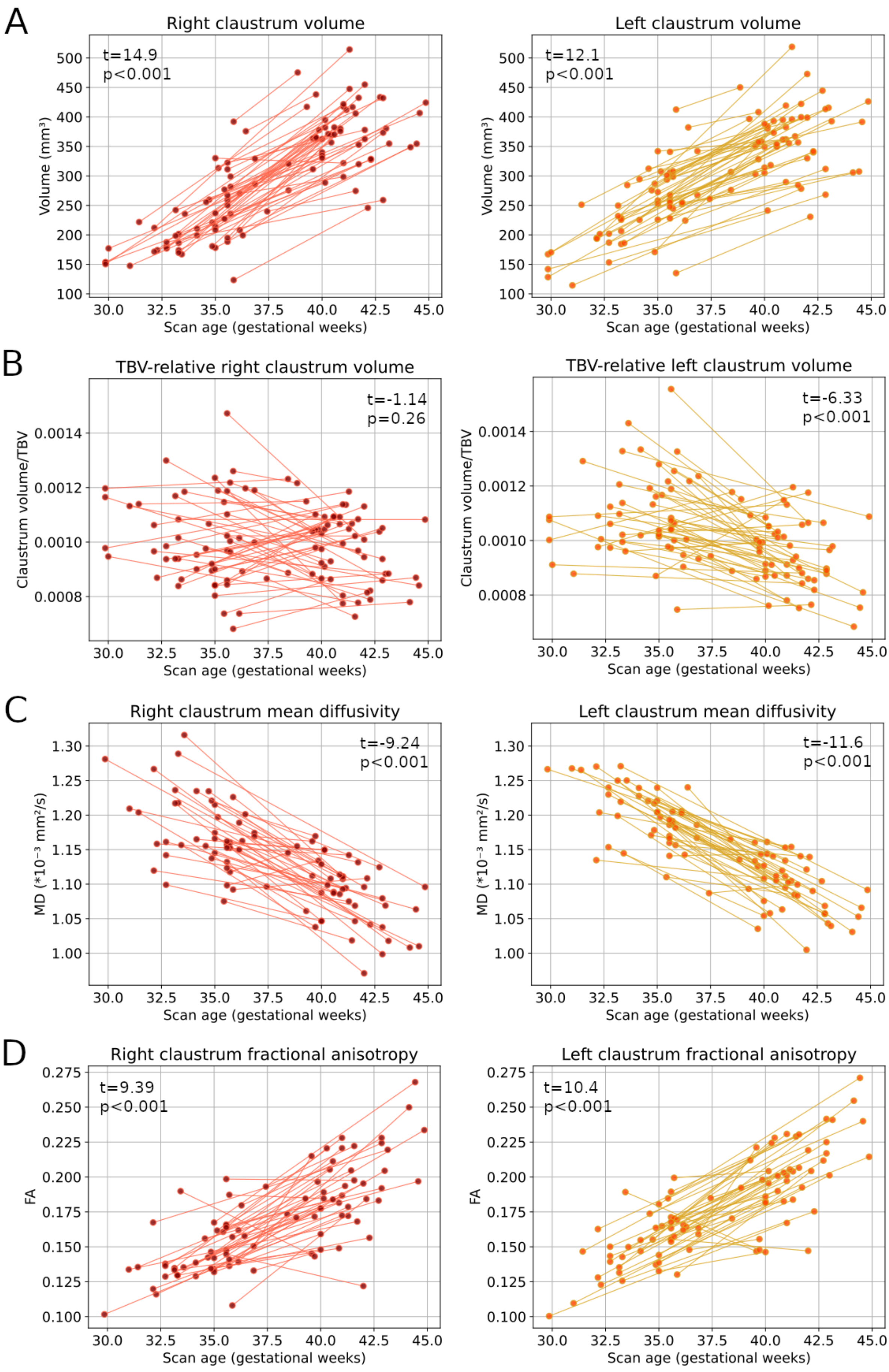
A
Right claustrum volume
t=14.9
p<0.001
Volume (mm³)
Scan age (gestational weeks)
Left claustrum volume
t=12.1
p<0.001
B
TBV-relative right claustrum volume
t=-1.14
p=0.26
Claustrum volume/TBV
TBV-relative left claustrum volume
t=-6.33
p<0.001
C
Right claustrum mean diffusivity
t=-9.24
p<0.001
MD (*10⁻³ mm²/s)
Left claustrum mean diffusivity
t=-11.6
p<0.001
D
Right claustrum fractional anisotropy
t=9.39
p<0.001
FA
Left claustrum fractional anisotropy
t=10.4
p<0.001

Fig. S2: Claustrum development after birth of preterm-born neonates for right and left claustrum separately. Macrostructure: (A) Longitudinal examination of the claustrum structure development in preterm-born subjects. The absolute claustrum volume rose and (B) the total brain volume (TBV-) relative claustrum volume decreased in the postnatal period of 53 preterm-born subjects. Microstructure: (C) The claustrum mean diffusivity (MD) decreased in the first weeks after preterm birth, while (D) the claustrum fractional anisotropy (FA) rose, shown in 45 subjects. T-statistics and p-values were calculated by a paired t-test between the first and second scans, respectively.

Table S4: Demographic characteristics of term-born neonates of the developing Human Connectome Project, second data release, with T2-weighted scans, who were included in the comparison analysis preterm-vs.-term, and who were not included. SD=standard deviation.

	Subjects (Male)	Singleton status (Singles / Multiples)	Birth age (Mean ± SD)	Birth weight (kg)
Term-born neonates included in the analysis preterm-vs.-term	83 (48)	81 / 2	39.9 (±1.2)	3.34 (±0.56)
Term-born neonates who were **not** included	295 (157)	280 / 15	40.0 (±1.2)	3.38 (±0.51)

Table S5: Demographic characteristics of term-born neonates of the developing Human Connectome Project, second data release, with diffusion-weighted scans, who were included in the comparison analysis preterm-vs.-term, and who were not included. SD=standard deviation.

	Subjects (Male)	Singleton status (Singles / Multiples)	Birth age (Mean ± SD)	Birth weight (kg)

Term-born neonates included in the analysis preterm-vs.-term	72 (41)	70 / 2	39.8 (±1.2)	3.31 (±0.57)
Term-born neonates who were **not** included	255 (134)	240 / 15	39.9 (±1.2)	3.35 (±0.52)

Table S6: Comparison of preterm- (PT) and full term-born (FT) neonates. For this analysis, all available term-born subjects of the dHCP, second data release, were included as controls instead of selecting a specific age matched sample. That comprises 377 T2-weighted scans and 326 diffusion-weighted scans of term-born neonates. While the metrics absolute and relative claustrum volume, mean diffusivity (MD), and fractional anisotropy (FA) were the dependent variables in general linear models, respectively, preterm birth was the independent variable, corrected for scan age and sex. SD=standard deviation, TBV=total brain volume.

Metric	FT: Mean (± SD)	PT: Mean (± SD)	p-value	partial η^2
Mean claustrum volume (mm^3)	330 (±50)	360 (±55)	<0.001	0.05
Mean TBV-relative claustrum volume	0.00184 (±0.00023)	0.00195 (±0.00025)	<0.001	0.04
Mean claustrum MD (10^{-3} mm^2/s)	1.08 (±0.04)	1.10 (±0.04)	<0.001	0.04
Mean claustrum FA	0.216 (±0.027)	0.193 (±0.027)	<0.001	0.13

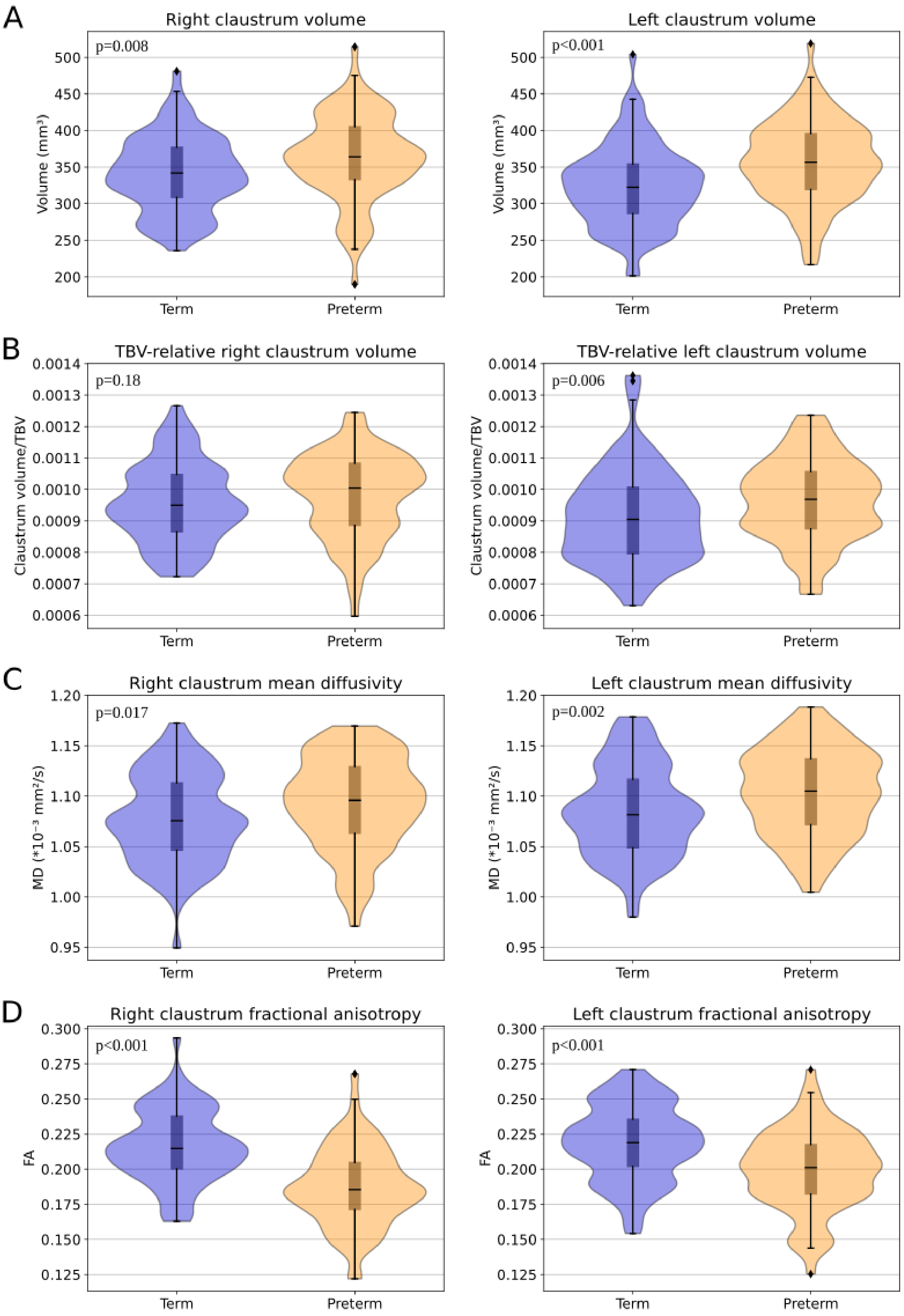

Fig. S3: Impact of preterm birth on right and left claustrum structure. We compared claustrum structure between groups of preterm- and term-born neonates for right and left claustrum

separately. Significant differences ($p<0.05$) between groups were tested with general linear models correcting for scan age and sex for each metric, respectively. Macrostructure: (A) The absolute claustrum volume and (B) the claustrum volume in relation to the total brain volume (TBV) were significantly increased after preterm birth tested in 83 preterm- and 83 term-born neonates, respectively, at term-equivalent age. Microstructure: (C) The claustrum mean diffusivity (MD) was increased in preterm-born neonates while (D) the claustrum fractional anisotropy (FA) was decreased in preterm-born neonates tested on 72 preterm- and 72 term-born neonates, respectively, at term-equivalent age.

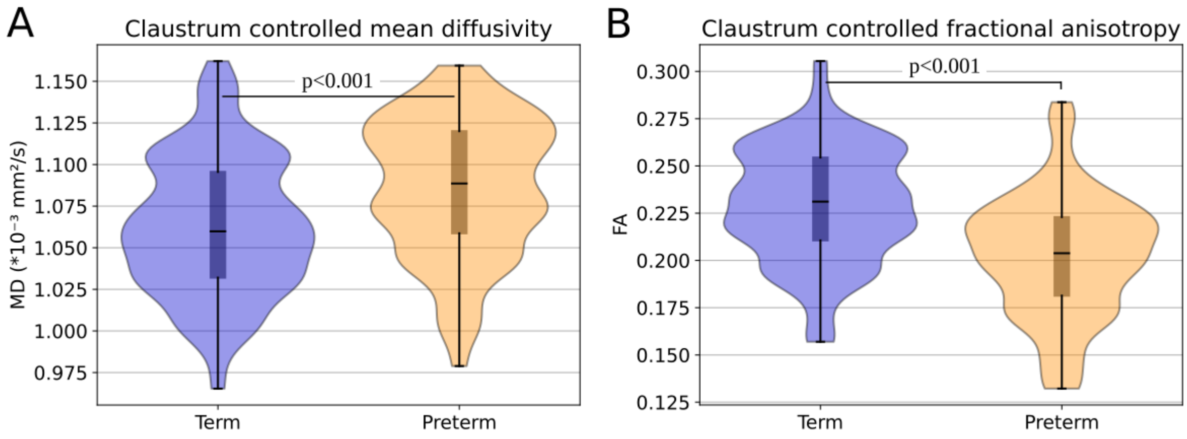

Fig. S4: Impact of preterm birth on claustrum-controlled microstructure. Claustrum-controlled segmentation was defined as claustrum voxels that are surrounded by other claustrum voxels for at least 90%. Significant differences ($p<0.05$) between preterm- and term-born neonates were tested with general linear models correcting for scan age and sex, respectively. (A) The claustrum-controlled mean diffusivity (MD) was significantly increased in preterm-born neonates (preterm-born subjects: 1.09 ± 0.04 $*10^{-3}$ mm^2/s, term-born subjects: 1.06 ± 0.04 $*10^{-3}$ mm^2/s; $p<0.0001$; partial $\eta^2 = 0.11$) while (B) the claustrum-controlled fractional anisotropy (FA) was significantly decreased in preterm-born neonates (preterm-born subjects: 0.203 ± 0.033, term-born subjects: 0.230 ± 0.031; $p<0.0001$; partial $\eta^2 = 0.17$) tested on 72 preterm- and term-born neonates, respectively, at term-equivalent age.

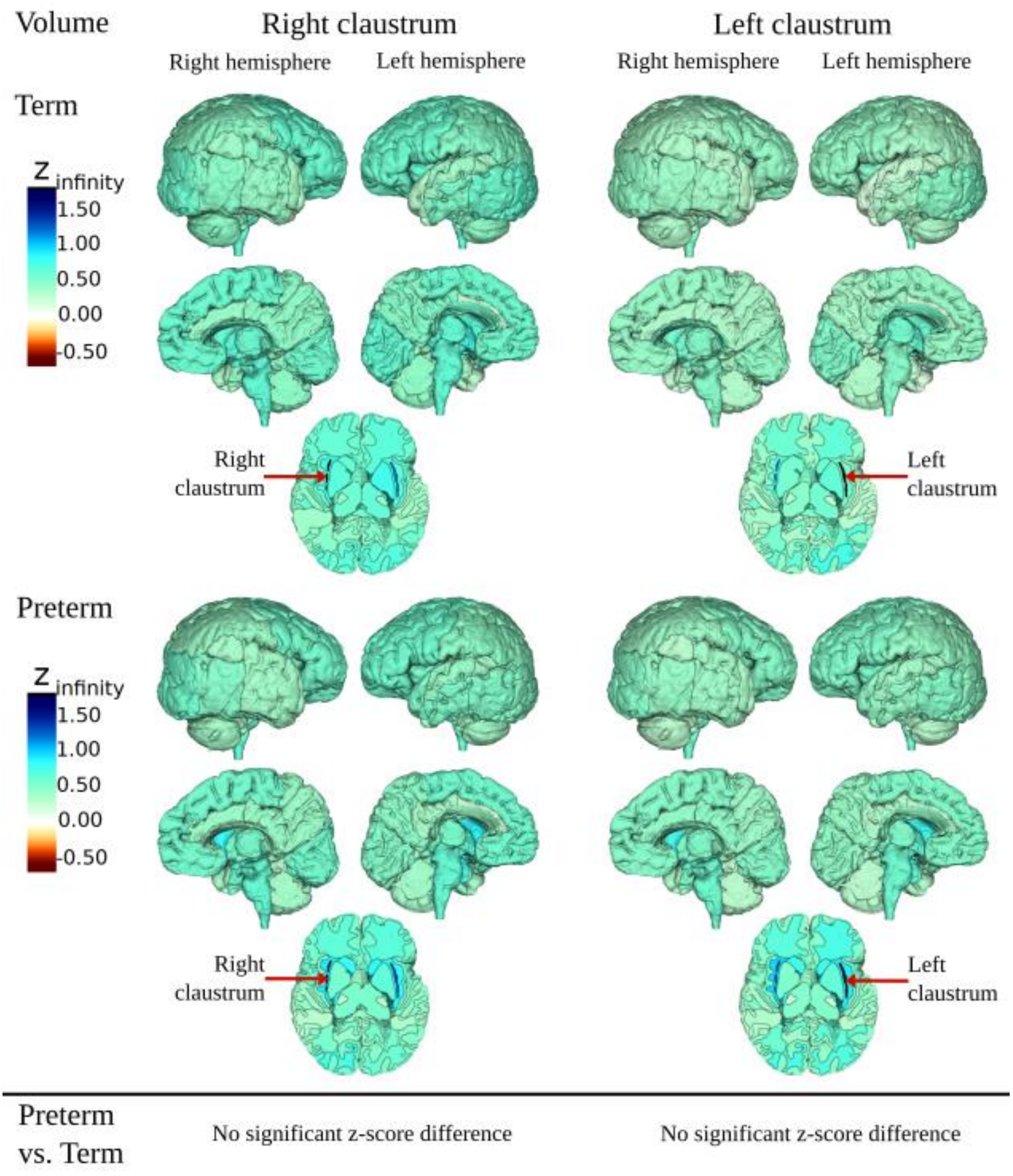

Fig. S5: Correlation of the claustrum volume with volumes of cortical and subcortical gray and white matter regions. The analysis was performed separately for the right and left claustrum. Pearson's r correlation coefficients were calculated for age-equivalent groups of 83 preterm- and 83 term-born neonates, respectively, and Fisher z-transformed. All correlations were positive in this young cohort with growing brains. There was no significant z-score difference between the volume correlations in preterm- and term-born neonates.

Table S7: Z-scored correlation coefficients between absolute claustrum volume and the absolute volume of the regions defined in the Draw-EM atlas in preterm- and term-born neonates for right and left claustrum separately. To test for significant differences, the p-values were calculated for all regions and corrected for multiple testing with false discovery rate correction (p_FDR). There is no significant ($p<0.05$) difference. Ant. = anterior, cla = claustrum, FT = full term, GM = gray matter, inf. = inferior, lat. = lateral, lh = left hemisphere, med. = medial, occ. = occipital, post. = posterior, PT = preterm, rh = right hemisphere, sup. = superior, temp. = temporal, WM = white matter

Region	PT (right cla)	FT (right cla)	p_FDR (right cla)	PT (left cla)	FT (left cla)	p_FDR (left cla)
lh hippocampus	0.527	0.465	0.986	0.486	0.341	0.965
rh hippocampus	0.523	0.418	0.986	0.500	0.319	0.965
lh amygdala	0.753	0.599	0.986	0.738	0.507	0.965
rh amygdala	0.663	0.648	0.986	0.616	0.532	0.965
lh ant. temp. med. GM	0.363	0.269	0.986	0.311	0.156	0.965
rh ant. temp. med. GM	0.338	0.324	0.986	0.309	0.233	0.965
lh ant. temp. lat. GM	0.511	0.289	0.986	0.421	0.173	0.965
rh ant. temp. lat. GM	0.368	0.353	0.986	0.328	0.255	0.965
lh ant. parahippocampal GM	0.726	0.363	0.966	0.656	0.256	0.829
rh ant. parahippocampal GM	0.566	0.424	0.986	0.494	0.328	0.965
lh sup. temp. middle gyrus GM	0.423	0.380	0.986	0.384	0.229	0.965
rh sup. temp. middle gyrus GM	0.372	0.441	0.986	0.361	0.275	0.965
lh med.-inf. temp. ant. gyrus GM	0.495	0.502	0.986	0.424	0.343	0.965
rh med.-inf. temp. ant. gyrus GM	0.370	0.412	0.986	0.376	0.358	0.965
lh lat. occ.-temp. ant. gyrus GM	0.434	0.290	0.986	0.313	0.257	0.965
rh lat. occ.-temp. ant. gyrus GM	0.465	0.393	0.986	0.394	0.256	0.965
lh cerebellum	0.366	0.443	0.986	0.283	0.310	0.965
rh cerebellum	0.357	0.445	0.986	0.283	0.312	0.965
brainstem	0.612	0.618	0.986	0.571	0.469	0.965
rh insula GM	0.508	0.571	0.986	0.532	0.427	0.965
lh insula GM	0.528	0.601	0.986	0.548	0.469	0.965
rh occipital GM	0.547	0.555	0.986	0.473	0.419	0.965
lh occipital GM	0.479	0.611	0.986	0.432	0.495	0.965
rh post. parahippocampal GM	0.282	0.430	0.986	0.253	0.360	0.965
lh post. parahippocampal GM	0.389	0.338	0.986	0.342	0.269	0.965
rh lat. occ.-temp. post. gyrus GM	0.462	0.295	0.986	0.380	0.203	0.965
lh lat. occ.-temp. post. gyrus GM	0.432	0.360	0.986	0.364	0.235	0.965
rh med.-inf. temp. post. gyrus GM	0.470	0.461	0.986	0.411	0.386	0.965
lh med.-inf. temp. post. gyrus GM	0.491	0.567	0.986	0.472	0.448	0.965

rh sup. temp. post. gyrus GM	0.351	0.393	0.986	0.294	0.313	0.965
lh sup. temp. post. gyrus GM	0.360	0.365	0.986	0.338	0.245	0.965
rh ant. cingulate gyrus GM	0.399	0.398	0.995	0.435	0.321	0.965
lh ant. cingulate gyrus GM	0.202	0.235	0.986	0.270	0.199	0.965
rh post. cingulate gyrus GM	0.412	0.436	0.986	0.437	0.308	0.965
lh post. cingulate gyrus GM	0.351	0.500	0.986	0.324	0.380	0.965
rh frontal GM	0.541	0.576	0.986	0.470	0.408	0.965
lh frontal GM	0.564	0.574	0.986	0.492	0.405	0.965
rh parietal GM	0.398	0.440	0.986	0.377	0.331	0.965
lh parietal GM	0.453	0.466	0.986	0.414	0.329	0.965
rh caudate nucleus	0.805	0.623	0.986	0.764	0.551	0.965
lh caudate nucleus	0.835	0.654	0.986	0.770	0.578	0.965
rh thalamus high T2 intensity	0.532	0.585	0.986	0.490	0.481	0.985
lh thalamus high T2 intensity	0.529	0.616	0.986	0.502	0.562	0.965
rh subthalamic nucleus	0.462	0.572	0.986	0.476	0.474	0.992
lh subthalamic nucleus	0.537	0.642	0.986	0.528	0.513	0.968
rh lentiform nucleus	0.542	0.654	0.986	0.512	0.554	0.965
lh lentiform nucleus	0.636	0.736	0.986	0.616	0.624	0.985
corpus callosum	0.396	0.551	0.986	0.431	0.507	0.965
lh ant. temp. med. WM	0.247	0.142	0.986	0.200	0.032	0.965
rh ant. temp. med. WM	0.220	0.167	0.986	0.222	0.099	0.965
lh ant. temp. lat. WM	0.474	0.289	0.986	0.321	0.159	0.965
rh ant. temp. lat. WM	0.379	0.347	0.986	0.285	0.251	0.965
lh ant. parahippocampal WM	0.478	0.373	0.986	0.280	0.282	0.992
rh ant. parahippocampal WM	0.390	0.359	0.986	0.279	0.336	0.965
lh sup. temp. middle gyrus WM	0.493	0.361	0.986	0.484	0.353	0.965
rh sup. temp. middle gyrus WM	0.451	0.589	0.986	0.506	0.547	0.965
lh med.-inf. temp. ant. gyrus WM	0.488	0.382	0.986	0.448	0.319	0.965
rh med.-inf. temp. ant. gyrus WM	0.418	0.280	0.986	0.449	0.426	0.965
lh lat. occ.-temp. ant. gyrus WM	0.353	0.099	0.986	0.214	0.096	0.965
rh lat. occ.-temp. ant. gyrus WM	0.410	0.289	0.986	0.317	0.232	0.965
rh insula WM	0.875	0.746	0.986	0.941	0.621	0.829
lh insula WM	0.818	0.661	0.986	0.883	0.539	0.829
rh occipital WM	0.723	0.657	0.986	0.717	0.608	0.965
lh occipital WM	0.588	0.669	0.986	0.688	0.733	0.965
rh post. parahippocampal WM	0.545	0.452	0.986	0.491	0.405	0.965
lh post. parahippocampal WM	0.476	0.309	0.986	0.386	0.315	0.965
rh lat. occ.-temp. post. gyrus WM	0.438	0.464	0.986	0.407	0.432	0.965
lh lat. occ.-temp. post. gyrus WM	0.583	0.390	0.986	0.567	0.314	0.965
rh med.-inf. temp. post. gyrus WM	0.648	0.426	0.986	0.713	0.569	0.965
lh med.-inf. temp. post. gyrus WM	0.471	0.525	0.986	0.629	0.678	0.965
rh sup. temp. post. gyrus WM	0.299	0.388	0.986	0.330	0.383	0.965
lh sup. temp. post. gyrus WM	0.337	0.394	0.986	0.290	0.317	0.965
rh ant. cingulate gyrus WM	0.505	0.398	0.986	0.587	0.327	0.965
lh ant. cingulate gyrus WM	0.335	0.285	0.986	0.332	0.244	0.965
rh post. cingulate gyrus WM	0.510	0.384	0.986	0.520	0.206	0.829
lh post. cingulate gyrus WM	0.504	0.474	0.986	0.436	0.342	0.965
rh frontal WM	0.635	0.609	0.986	0.661	0.593	0.965

lh frontal WM	0.656	0.650	0.986	0.707	0.625	0.965
rh parietal WM	0.525	0.482	0.986	0.592	0.538	0.965
lh parietal WM	0.539	0.519	0.986	0.620	0.569	0.965
rh thalamus low T2 intensity	0.262	0.395	0.986	0.215	0.301	0.965
lh thalamus low T2 intensity	0.271	0.412	0.986	0.220	0.242	0.965
rh claustrum	infinity	infinity	-	1.335	1.176	0.965
lh claustrum	1.335	1.176	0.986	infinity	infinity	-

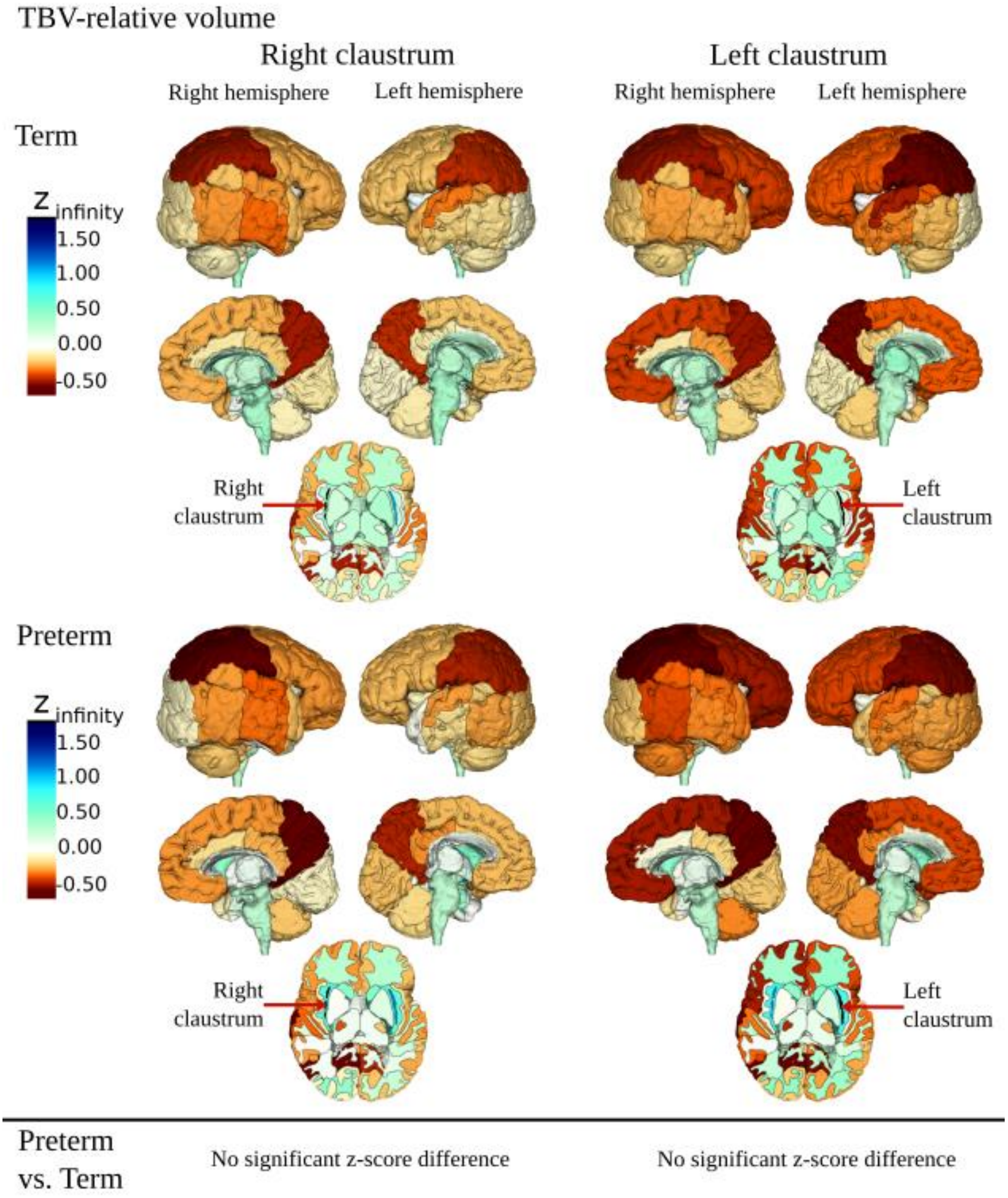

Fig. S6: Correlation of the total brain volume (TBV-)relative claustrum volume with TBV-relative volumes of cortical and subcortical gray and white matter regions. The analysis was performed separately for the right and left claustrum. Pearson's r correlation coefficients were calculated for age-equivalent groups of 83 preterm- and 83 term-born neonates, respectively, and Fisher z-transformed. While subcortical structures tended to have positive correlations, cortical gray matter showed mainly negative correlations with the claustrum. There was no significant z-score difference between the TBV-relative volume correlations in preterm- and term-born neonates.

Table S8: Z-scores correlation coefficients between total brain volume (TBV-)relative claustrum volume and the TBV-relative volume of the regions defined in the Draw-EM atlas in preterm- and term-born neonates for right and left claustrum separately. To test for significant differences, p-values were calculated for all regions and corrected for multiple testing with false discovery rate correction (p_FDR). There is no significant (p<0.05) difference. Ant. = anterior, cla = claustrum, FT = full term, GM = gray matter, inf. = inferior, lat. = lateral, lh = left hemisphere, med. = medial, occ. = occipital, post. = posterior, PT = preterm, rh = right hemisphere, sup. = superior, temp. = temporal, WM = white matter

Region	PT (right cla)	FT (right cla)	p_FDR (right cla)	PT (left cla)	FT (left cla)	p_FDR (left cla)
lh hippocampus	0.191	0.139	0.958	0.156	0.108	0.978
rh hippocampus	0.202	0.017	0.736	0.203	0.009	0.978
lh amygdala	0.402	0.292	0.881	0.440	0.290	0.978
rh amygdala	0.279	0.364	0.897	0.265	0.338	0.978
lh ant. temp. med. GM	-0.028	-0.147	0.881	-0.107	-0.206	0.978
rh ant. temp. med. GM	-0.106	-0.241	0.851	-0.164	-0.236	0.978
lh ant. temp. lat. GM	-0.048	-0.238	0.736	-0.210	-0.330	0.978
rh ant. temp. lat. GM	-0.239	-0.231	0.991	-0.329	-0.280	0.978
lh ant. parahippocampal GM	0.424	0.094	0.410	0.362	0.041	0.613
rh ant. parahippocampal GM	0.141	0.066	0.924	0.064	0.043	0.984
lh sup. temp. middle gyrus GM	-0.291	-0.338	0.958	-0.345	-0.475	0.978
rh sup. temp. middle gyrus GM	-0.375	-0.298	0.924	-0.353	-0.462	0.978
lh med.-inf. temp. ant. gyrus GM	-0.167	-0.185	0.991	-0.307	-0.324	0.984
rh med.-inf. temp. ant. gyrus GM	-0.346	-0.357	0.991	-0.325	-0.272	0.978
lh lat. occ.-temp. ant. gyrus GM	0.103	-0.182	0.480	-0.039	-0.154	0.978

rh lat. occ.-temp. ant. gyrus GM	-0.035	-0.132	0.881	-0.127	-0.212	0.978
lh cerebellum	-0.184	-0.126	0.958	-0.297	-0.200	0.978
rh cerebellum	-0.223	-0.109	0.881	-0.320	-0.178	0.978
brainstem	0.258	0.344	0.897	0.259	0.283	0.978
rh insula GM	-0.123	-0.012	0.881	-0.058	-0.070	0.987
lh insula GM	-0.108	0.054	0.813	-0.053	0.007	0.978
rh occipital GM	-0.095	-0.144	0.958	-0.211	-0.210	0.993
lh occipital GM	-0.231	-0.090	0.826	-0.288	-0.110	0.978
rh post. parahippocampal GM	-0.114	-0.073	0.958	-0.157	-0.090	0.978
lh post. parahippocampal GM	-0.056	-0.110	0.958	-0.125	-0.133	0.993
rh lat. occ.-temp. post. gyrus GM	-0.158	-0.194	0.976	-0.307	-0.234	0.978
lh lat. occ.-temp. post. gyrus GM	-0.038	-0.085	0.958	-0.136	-0.171	0.978
rh med.-inf. temp. post. gyrus GM	-0.302	-0.303	0.997	-0.409	-0.294	0.978
lh med.-inf. temp. post. gyrus GM	-0.276	-0.171	0.881	-0.285	-0.243	0.978
rh sup. temp. post. gyrus GM	-0.293	-0.225	0.937	-0.371	-0.228	0.978
lh sup. temp. post. gyrus GM	-0.195	-0.295	0.881	-0.213	-0.367	0.978
rh ant. cingulate gyrus GM	-0.167	-0.137	0.983	-0.074	-0.139	0.978
lh ant. cingulate gyrus GM	-0.221	-0.058	0.813	-0.081	-0.064	0.984
rh post. cingulate gyrus GM	-0.261	-0.214	0.958	-0.185	-0.288	0.978
lh post. cingulate gyrus GM	-0.305	-0.126	0.753	-0.327	-0.200	0.978
rh frontal GM	-0.299	-0.256	0.958	-0.489	-0.424	0.978
lh frontal GM	-0.236	-0.222	0.991	-0.411	-0.384	0.978
rh parietal GM	-0.593	-0.493	0.881	-0.594	-0.501	0.978
lh parietal GM	-0.469	-0.453	0.991	-0.545	-0.558	0.987
rh caudate nucleus	0.454	0.244	0.736	0.458	0.255	0.978
lh caudate nucleus	0.500	0.268	0.688	0.465	0.281	0.978
rh thalamus high T2 intensity	0.084	0.231	0.826	0.085	0.270	0.978
lh thalamus high T2 intensity	0.071	0.272	0.736	0.096	0.403	0.658
rh subthalamic nucleus	0.041	0.232	0.736	0.094	0.192	0.978
lh subthalamic nucleus	0.083	0.359	0.480	0.107	0.267	0.978
rh lentiform nucleus	-0.026	0.202	0.688	-0.044	0.186	0.967
lh lentiform nucleus	0.143	0.331	0.736	0.161	0.300	0.978
corpus callosum	0.066	0.253	0.736	0.134	0.230	0.978
lh ant. temp. med. WM	-0.070	-0.166	0.881	-0.139	-0.220	0.978
rh ant. temp. med. WM	-0.069	-0.228	0.813	-0.060	-0.205	0.978
lh ant. temp. lat. WM	-0.002	-0.209	0.736	-0.236	-0.308	0.978
rh ant. temp. lat. WM	-0.117	-0.108	0.991	-0.266	-0.154	0.978
lh ant. parahippocampal WM	0.413	0.064	0.410	0.192	0.031	0.978
rh ant. parahippocampal WM	0.333	0.091	0.688	0.217	0.169	0.978
lh sup. temp. middle gyrus WM	0.243	0.102	0.826	0.257	0.136	0.978
rh sup. temp. middle gyrus WM	0.174	0.320	0.826	0.280	0.321	0.978
lh med.-inf. temp. ant. gyrus WM	0.099	0.186	0.897	0.060	0.178	0.978
rh med.-inf. temp. ant. gyrus WM	-0.007	0.064	0.929	0.065	0.291	0.967
lh lat. occ.-temp. ant. gyrus WM	0.030	-0.270	0.480	-0.158	-0.221	0.978
rh lat. occ.-temp. ant. gyrus WM	0.120	0.001	0.881	-0.000	0.024	0.978
rh insula WM	0.664	0.307	0.410	0.761	0.270	0.083
lh insula WM	0.630	0.233	0.410	0.730	0.189	0.055
rh occipital WM	0.454	0.170	0.480	0.464	0.210	0.947

lh occipital WM	0.224	0.224	0.997	0.382	0.416	0.978
rh post. parahippocampal WM	0.466	0.180	0.480	0.399	0.163	0.967
lh post. parahippocampal WM	0.242	0.112	0.858	0.119	0.167	0.978
rh lat. occ.-temp. post. gyrus WM	0.154	0.185	0.983	0.130	0.196	0.978
lh lat. occ.-temp. post. gyrus WM	0.318	-0.033	0.410	0.308	-0.061	0.432
rh med.-inf. temp. post. gyrus WM	0.318	0.245	0.924	0.439	0.436	0.993
lh med.-inf. temp. post. gyrus WM	0.107	0.275	0.813	0.372	0.470	0.978
rh sup. temp. post. gyrus WM	-0.033	0.008	0.958	0.046	0.074	0.978
lh sup. temp. post. gyrus WM	0.176	-0.024	0.736	0.151	-0.032	0.978
rh ant. cingulate gyrus WM	0.253	0.022	0.688	0.371	0.022	0.483
lh ant. cingulate gyrus WM	0.226	0.005	0.713	0.263	-0.006	0.863
rh post. cingulate gyrus WM	0.111	0.006	0.881	0.170	-0.117	0.769
lh post. cingulate gyrus WM	0.209	0.065	0.826	0.133	-0.009	0.978
rh frontal WM	0.279	0.265	0.991	0.371	0.344	0.978
lh frontal WM	0.297	0.300	0.997	0.423	0.377	0.978
rh parietal WM	0.056	0.084	0.983	0.234	0.268	0.978
lh parietal WM	0.111	0.128	0.991	0.298	0.299	0.993
rh thalamus low T2 intensity	-0.396	-0.019	0.410	-0.474	-0.085	0.407
lh thalamus low T2 intensity	-0.220	0.121	0.410	-0.286	-0.043	0.967
rh claustrum	infinity	infinity	-	1.141	1.054	0.978
lh claustrum	1.141	1.054	0.897	infinity	infinity	-

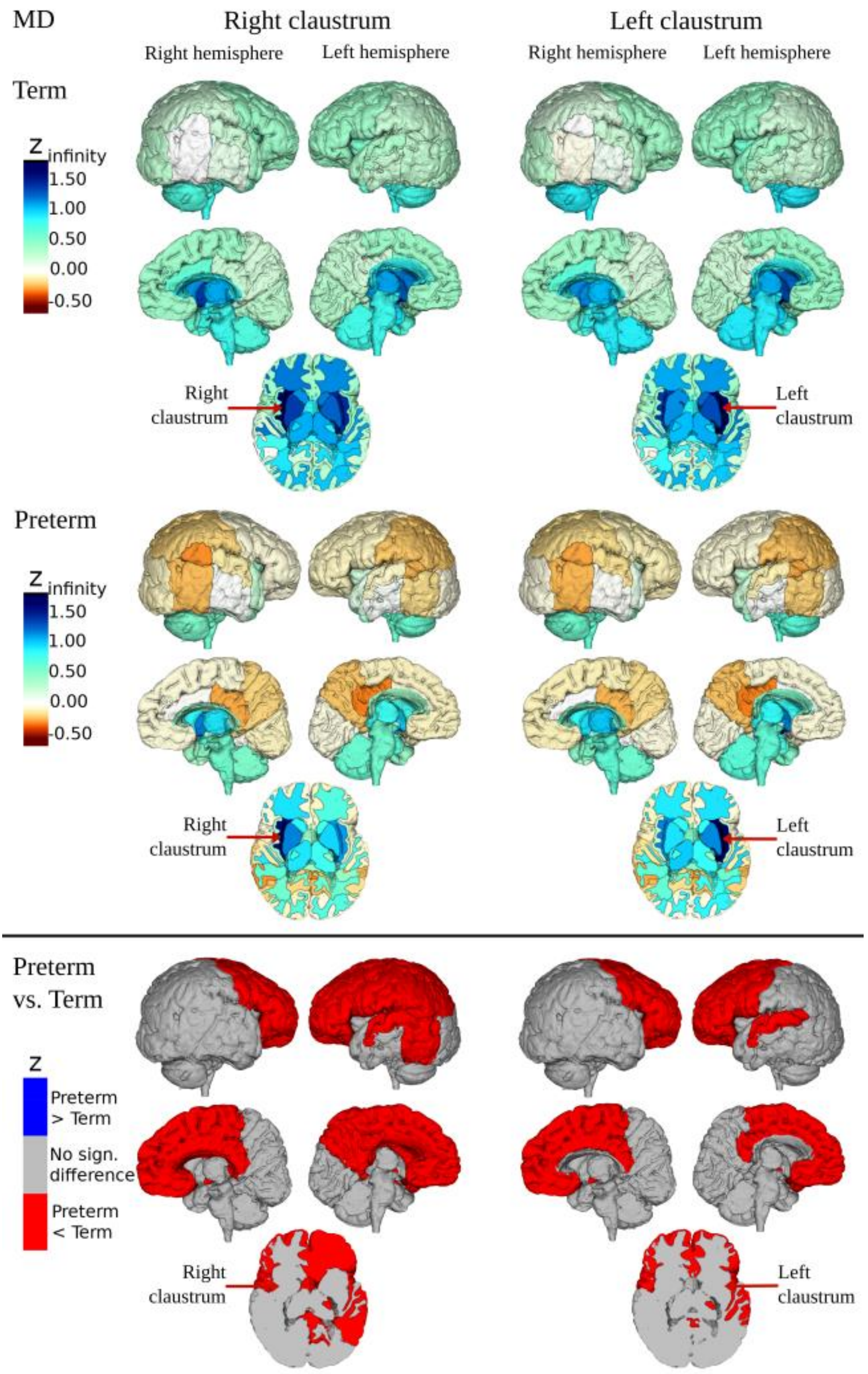
MD
Right claustrum
Left claustrum
Right hemisphere
Left hemisphere
Right hemisphere
Left hemisphere
Term
Z
infinity
1.50
1.00
0.50
0.00
-0.50
Right claustrum
Left claustrum
Preterm
Z
infinity
1.50
1.00
0.50
0.00
-0.50
Right claustrum
Left claustrum
Preterm vs. Term
Z
Preterm > Term
No sign. difference
Preterm < Term
Right claustrum
Left claustrum

Fig. S7: Correlation of the claustrum mean diffusivity (MD) with MD of cortical and subcortical gray and white matter regions. The analysis was performed separately for the right and left claustrum. Pearson's r correlation coefficients were calculated for age-equivalent groups of 72 preterm- and 72 term-born neonates, respectively, and Fisher z-transformed. While subcortical structures tended to have high positive correlations, cortical gray matter showed lower positive correlations with the claustrum in term-born neonates and no to negative correlations with the claustrum in preterm-born neonates. The lower part presents regions with significantly different z-scores after premature birth.

Table S9: Z-scored correlation coefficients between claustrum mean diffusivity and the mean diffusivity of the regions defined in the Draw-EM atlas in preterm- and term-born neonates for right and left claustrum separately. To test for significant differences, p-values were calculated for all regions and corrected for multiple testing with false discovery rate correction (p_FDR). Significant p-values (p<0.05) are labeled with a *. Ant. = anterior, cla = claustrum, FT = full term, GM = gray matter, inf. = inferior, lat. = lateral, lh = left hemisphere, med. = medial, occ. = occipital, post. = posterior, PT = preterm, rh = right hemisphere, sup. = superior, temp. = temporal, WM = white matter

Region	PT (right cla)	FT (right cla)	p_FDR (right cla)	PT (left cla)	FT (left cla)	p_FDR (left cla)
lh hippocampus	0.741	0.773	0.850	0.892	0.897	0.977
rh hippocampus	0.564	0.791	0.213	0.447	0.738	0.167
lh amygdala	0.614	1.068	0.030*	0.741	1.132	0.068
rh amygdala	0.666	0.806	0.426	0.622	0.734	0.540
lh ant. temp. med. GM	0.397	0.669	0.140	0.507	0.757	0.239
rh ant. temp. med. GM	0.386	0.593	0.252	0.451	0.638	0.309
lh ant. temp. lat. GM	0.134	0.313	0.314	0.206	0.439	0.244
rh ant. temp. lat. GM	0.216	0.327	0.526	0.097	0.280	0.320
lh ant. parahippocampal GM	0.662	0.930	0.141	0.719	0.946	0.244
rh ant. parahippocampal GM	0.428	0.739	0.098	0.508	0.823	0.152
lh sup. temp. middle gyrus GM	-0.088	0.317	0.045*	-0.125	0.353	0.027*
rh sup. temp. middle gyrus GM	-0.132	0.193	0.084	-0.156	0.200	0.109
lh med.-inf. temp. ant. gyrus GM	0.042	0.222	0.314	0.031	0.233	0.285
rh med.-inf. temp. ant. gyrus GM	0.032	0.108	0.655	-0.030	0.067	0.601

lh lat. occ.-temp. ant. gyrus GM	0.479	0.749	0.140	0.541	0.721	0.327
rh lat. occ.-temp. ant. gyrus GM	0.244	0.609	0.070	0.235	0.656	0.055
lh cerebellum	0.589	0.740	0.394	0.650	0.878	0.244
rh cerebellum	0.507	0.693	0.302	0.575	0.856	0.174
brainstem	0.558	0.772	0.237	0.635	0.864	0.244
rh insula GM	0.391	0.515	0.476	0.241	0.440	0.285
lh insula GM	0.187	0.517	0.082	0.250	0.556	0.155
rh occipital GM	-0.100	0.262	0.070	-0.058	0.250	0.154
lh occipital GM	-0.063	0.286	0.071	-0.064	0.245	0.154
rh post. parahippocampal GM	-0.027	0.327	0.071	-0.004	0.338	0.122
lh post. parahippocampal GM	0.050	0.341	0.118	0.095	0.399	0.155
rh lat. occ.-temp. post. gyrus GM	-0.047	0.351	0.045*	-0.039	0.311	0.112
lh lat. occ.-temp. post. gyrus GM	0.316	0.423	0.535	0.411	0.429	0.922
rh med.-inf. temp. post. gyrus GM	-0.239	-0.011	0.212	-0.247	-0.051	0.288
lh med.-inf. temp. post. gyrus GM	-0.171	0.237	0.045*	-0.204	0.193	0.067
rh sup. temp. post. gyrus GM	-0.305	-0.005	0.106	-0.283	-0.009	0.182
lh sup. temp. post. gyrus GM	-0.250	0.240	0.023*	-0.273	0.234	0.024*
rh ant. cingulate gyrus GM	-0.030	0.604	0.003*	-0.011	0.637	0.004*
lh ant. cingulate gyrus GM	-0.074	0.552	0.003*	-0.000	0.579	0.011*
rh post. cingulate gyrus GM	-0.271	0.215	0.023*	-0.250	0.195	0.039*
lh post. cingulate gyrus GM	-0.328	0.171	0.023*	-0.327	0.146	0.027*
rh frontal GM	-0.067	0.383	0.030*	-0.105	0.357	0.030*
lh frontal GM	-0.104	0.378	0.023*	-0.074	0.402	0.027*
rh parietal GM	-0.187	0.180	0.070	-0.174	0.154	0.139
lh parietal GM	-0.224	0.229	0.030*	-0.227	0.190	0.055
rh caudate nucleus	0.839	1.113	0.138	0.823	1.121	0.159
lh caudate nucleus	0.746	1.156	0.045*	0.812	1.223	0.055
rh thalamus high T2 intensity	0.902	1.051	0.394	0.976	1.023	0.821
lh thalamus high T2 intensity	0.765	1.066	0.106	0.812	1.046	0.244
rh subthalamic nucleus	0.382	0.890	0.023*	0.420	0.899	0.027*
lh subthalamic nucleus	0.250	0.855	0.004*	0.266	0.807	0.018*
rh lentiform nucleus	1.149	1.353	0.255	0.907	1.115	0.273
lh lentiform nucleus	0.948	1.235	0.118	1.132	1.304	0.340
corpus callosum	0.386	0.817	0.036*	0.500	0.822	0.143
lh ant. temp. med. WM	0.596	0.994	0.045*	0.730	1.066	0.129
rh ant. temp. med. WM	0.633	0.820	0.302	0.638	0.812	0.337
lh ant. temp. lat. WM	0.613	0.842	0.212	0.723	0.901	0.330
rh ant. temp. lat. WM	0.623	0.710	0.611	0.543	0.627	0.654
lh ant. parahippocampal WM	0.879	0.977	0.568	1.121	1.089	0.880
rh ant. parahippocampal WM	0.713	0.886	0.332	0.703	0.921	0.247
lh sup. temp. middle gyrus WM	0.711	1.084	0.067	0.940	1.163	0.247
rh sup. temp. middle gyrus WM	0.889	1.176	0.118	0.842	1.088	0.240
lh med.-inf. temp. ant. gyrus WM	0.587	0.939	0.071	0.774	1.021	0.240
rh med.-inf. temp. ant. gyrus WM	0.675	0.932	0.159	0.537	0.827	0.167
lh lat. occ.-temp. ant. gyrus WM	0.729	1.078	0.071	0.863	1.094	0.244
rh lat. occ.-temp. ant. gyrus WM	0.642	1.072	0.036*	0.593	1.007	0.055
rh insula WM	1.545	1.695	0.394	1.140	1.360	0.247
lh insula WM	1.079	1.403	0.084	1.642	1.620	0.906

rh occipital WM	0.676	1.018	0.077	0.810	1.036	0.245
lh occipital WM	0.684	0.994	0.099	0.844	0.986	0.428
rh post. parahippocampal WM	0.682	0.914	0.212	0.684	0.967	0.174
lh post. parahippocampal WM	0.668	0.833	0.355	0.841	1.008	0.352
rh lat. occ.-temp. post. gyrus WM	0.789	1.076	0.118	0.717	0.993	0.182
lh lat. occ.-temp. post. gyrus WM	0.693	1.055	0.070	0.902	1.122	0.247
rh med.-inf. temp. post. gyrus WM	0.718	1.047	0.082	0.669	0.903	0.244
lh med.-inf. temp. post. gyrus WM	0.581	0.999	0.042*	0.780	1.014	0.244
rh sup. temp. post. gyrus WM	0.637	0.763	0.473	0.654	0.683	0.887
lh sup. temp. post. gyrus WM	0.433	0.989	0.010*	0.611	1.000	0.068
rh ant. cingulate gyrus WM	0.606	0.956	0.071	0.658	0.897	0.244
lh ant. cingulate gyrus WM	0.435	0.775	0.077	0.682	0.900	0.247
rh post. cingulate gyrus WM	0.536	0.890	0.071	0.531	0.853	0.143
lh post. cingulate gyrus WM	0.411	0.811	0.045*	0.611	0.896	0.173
rh frontal WM	0.907	1.168	0.152	0.892	1.057	0.353
lh frontal WM	0.688	1.126	0.035*	0.912	1.103	0.301
rh parietal WM	0.566	0.902	0.079	0.618	0.818	0.285
lh parietal WM	0.581	0.916	0.079	0.756	0.905	0.406
rh thalamus low T2 intensity	0.729	0.946	0.234	0.709	0.977	0.192
lh thalamus low T2 intensity	0.422	0.875	0.030*	0.410	0.939	0.018*
rh claustrum	infinity	infinity	-	1.233	1.533	0.157
lh claustrum	1.233	1.533	0.106	infinity	infinity	-

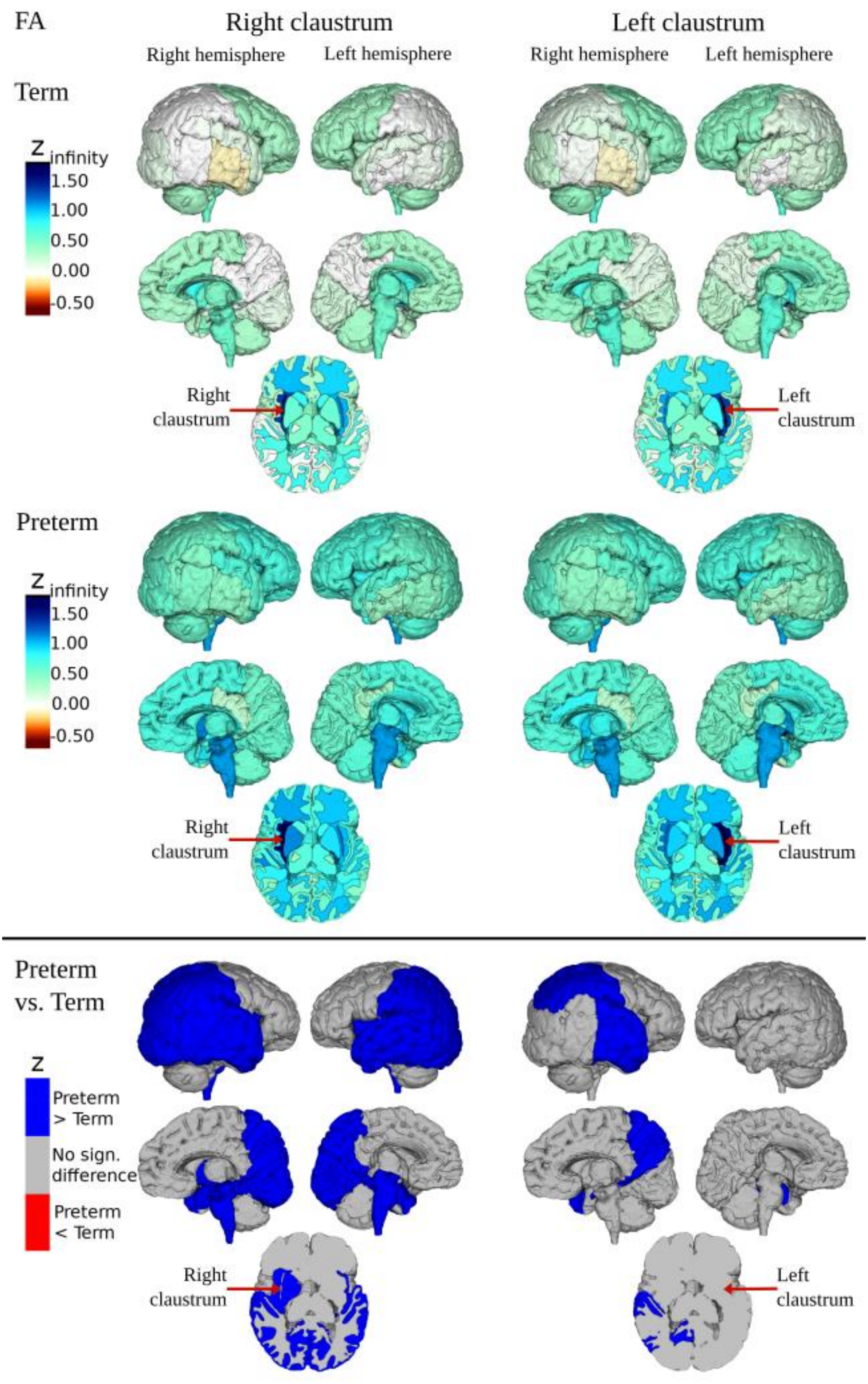
FA
Right claustrum
Left claustrum
Right hemisphere
Left hemisphere
Right hemisphere
Left hemisphere
Term
Z
infinity
1.50
1.00
0.50
0.00
-0.50
Right claustrum
Left claustrum
Preterm
Z
infinity
1.50
1.00
0.50
0.00
-0.50
Right claustrum
Left claustrum
Preterm vs. Term
Z
Preterm > Term
No sign. difference
Preterm < Term
Right claustrum
Left claustrum

Fig. S8: Correlation of the claustrum fractional anisotropy (FA) with FA of cortical and subcortical gray and white matter regions. The analysis was performed separately for the right and left claustrum. Pearson's r correlation coefficients were calculated for age-equivalent groups of 72 preterm- and 72 term-born neonates, respectively, and Fisher z-transformed. While subcortical structures tended to have high positive correlations, cortical gray matter showed lower positive correlations with the claustrum in preterm-born neonates and lower positive to no correlations with the claustrum in term-born neonates. The lower part presents regions with significantly different z-scores after premature birth.

Table S10: Z-scored correlation coefficients between claustrum fractional anisotropy and the fractional anisotropy of the regions defined in the Draw-EM atlas in preterm- and term-born neonates for right and left claustrum separately. To test for significant differences, p-values were calculated for all regions and corrected for multiple testing with false discovery rate correction (p_FDR). Significant p-values (p<0.05) are labeled with a *. Ant. = anterior, cla = claustrum, FT = full term, GM = gray matter, inf. = inferior, lat. = lateral, lh = left hemisphere, med. = medial, occ. = occipital, post. = posterior, PT = preterm, rh = right hemisphere, sup. = superior, temp. = temporal, WM = white matter

Region	PT (right cla)	FT (right cla)	p_FDR (right cla)	PT (left cla)	FT (left cla)	p_FDR (left cla)
lh hippocampus	0.628	0.089	0.006*	0.721	0.147	0.022*
rh hippocampus	0.795	0.217	0.006*	0.801	0.272	0.024*
lh amygdala	0.568	0.047	0.007*	0.581	0.119	0.044*
rh amygdala	0.703	0.163	0.006*	0.723	0.314	0.053
lh ant. temp. med. GM	0.769	0.291	0.013*	0.693	0.424	0.221
rh ant. temp. med. GM	0.736	0.262	0.013*	0.748	0.307	0.047*
lh ant. temp. lat. GM	0.611	0.148	0.015*	0.552	0.184	0.082
rh ant. temp. lat. GM	0.620	0.143	0.013*	0.578	0.126	0.047*
lh ant. parahippocampal GM	0.943	0.243	0.001*	0.972	0.421	0.022*
rh ant. parahippocampal GM	0.870	0.435	0.023*	0.966	0.580	0.068
lh sup. temp. middle gyrus GM	0.646	0.101	0.006*	0.613	0.191	0.053
rh sup. temp. middle gyrus GM	0.669	0.089	0.006*	0.605	0.142	0.044*

lh med.-inf. temp. ant. gyrus GM	0.388	0.011	0.046*	0.325	0.018	0.161
rh med.-inf. temp. ant. gyrus GM	0.441	-0.106	0.006*	0.423	-0.091	0.025*
lh lat. occ.-temp. ant. gyrus GM	0.423	0.061	0.054	0.319	0.098	0.333
rh lat. occ.-temp. ant. gyrus GM	0.444	0.216	0.237	0.380	0.184	0.337
lh cerebellum	0.656	0.354	0.118	0.665	0.346	0.141
rh cerebellum	0.569	0.332	0.224	0.634	0.400	0.292
brainstem	1.083	0.562	0.007*	1.068	0.659	0.053
rh insula GM	0.835	0.278	0.006*	0.677	0.300	0.075
lh insula GM	0.781	0.323	0.016*	0.865	0.454	0.053
rh occipital GM	0.587	0.105	0.013*	0.606	0.204	0.055
lh occipital GM	0.575	0.156	0.026*	0.531	0.226	0.161
rh post. parahippocampal GM	0.654	0.180	0.013*	0.651	0.290	0.082
lh post. parahippocampal GM	0.638	0.219	0.026*	0.581	0.376	0.337
rh lat. occ.-temp. post. gyrus GM	0.574	0.261	0.103	0.506	0.329	0.365
lh lat. occ.-temp. post. gyrus GM	0.602	0.180	0.026*	0.513	0.329	0.353
rh med.-inf. temp. post. gyrus GM	0.488	-0.001	0.012*	0.438	0.038	0.055
lh med.-inf. temp. post. gyrus GM	0.446	0.064	0.044*	0.420	0.137	0.208
rh sup. temp. post. gyrus GM	0.469	0.039	0.024*	0.456	0.127	0.127
lh sup. temp. post. gyrus GM	0.539	0.104	0.023*	0.537	0.173	0.082
rh ant. cingulate gyrus GM	0.723	0.438	0.138	0.796	0.577	0.333
lh ant. cingulate gyrus GM	0.754	0.511	0.214	0.789	0.548	0.273
rh post. cingulate gyrus GM	0.266	-0.009	0.151	0.295	0.099	0.337
lh post. cingulate gyrus GM	0.364	0.001	0.054	0.278	0.088	0.340
rh frontal GM	0.698	0.331	0.052	0.667	0.418	0.256
lh frontal GM	0.681	0.333	0.066	0.688	0.420	0.221
rh parietal GM	0.552	-0.003	0.006*	0.542	0.100	0.047*
lh parietal GM	0.556	0.033	0.007*	0.524	0.106	0.053
rh caudate nucleus	0.686	0.780	0.635	0.827	0.743	0.682
lh caudate nucleus	0.786	0.755	0.895	0.759	0.597	0.420
rh thalamus high T2 intensity	0.674	0.503	0.376	0.778	0.485	0.186
lh thalamus high T2 intensity	0.729	0.577	0.445	0.636	0.492	0.469
rh subthalamic nucleus	0.797	0.378	0.026*	0.831	0.469	0.082
lh subthalamic nucleus	0.761	0.461	0.119	0.590	0.380	0.337
rh lentiform nucleus	1.141	0.587	0.006*	0.970	0.553	0.053
lh lentiform nucleus	0.907	0.588	0.097	1.066	0.798	0.221
corpus callosum	0.751	0.485	0.166	0.733	0.527	0.337
lh ant. temp. med. WM	0.682	0.725	0.862	0.765	0.832	0.727
rh ant. temp. med. WM	0.756	0.705	0.857	0.830	0.777	0.794
lh ant. temp. lat. WM	0.707	0.595	0.579	0.765	0.690	0.696
rh ant. temp. lat. WM	0.635	0.599	0.891	0.781	0.589	0.340
lh ant. parahippocampal WM	0.868	0.574	0.126	1.059	0.778	0.209
rh ant. parahippocampal WM	1.063	0.654	0.030*	1.172	0.695	0.044*
lh sup. temp. middle gyrus WM	0.948	0.740	0.274	1.083	0.868	0.335
rh sup. temp. middle gyrus WM	1.120	0.836	0.138	0.967	0.768	0.337

lh med.-inf. temp. ant. gyrus WM	0.805	0.813	0.959	0.880	0.862	0.918
rh med.-inf. temp. ant. gyrus WM	0.804	0.707	0.634	0.871	0.671	0.337
lh lat. occ.-temp. ant. gyrus WM	0.674	0.660	0.949	0.689	0.642	0.810
rh lat. occ.-temp. ant. gyrus WM	0.683	0.705	0.929	0.733	0.649	0.682
rh insula WM	1.718	1.340	0.046*	1.160	1.062	0.635
lh insula WM	1.019	1.037	0.943	1.592	1.342	0.256
rh occipital WM	0.999	0.787	0.268	1.046	0.913	0.505
lh occipital WM	1.004	0.772	0.231	1.063	0.914	0.464
rh post. parahippocampal WM	0.927	0.601	0.088	0.937	0.748	0.340
lh post. parahippocampal WM	0.775	0.633	0.479	0.915	0.766	0.464
rh lat. occ.-temp. post. gyrus WM	0.917	0.706	0.269	0.788	0.642	0.465
lh lat. occ.-temp. post. gyrus WM	0.927	0.746	0.360	0.953	0.770	0.353
rh med.-inf. temp. post. gyrus WM	0.968	0.746	0.248	0.853	0.721	0.505
lh med.-inf. temp. post. gyrus WM	0.803	0.794	0.959	0.893	0.762	0.505
rh sup. temp. post. gyrus WM	0.848	0.707	0.479	0.770	0.673	0.635
lh sup. temp. post. gyrus WM	0.761	0.621	0.479	0.799	0.718	0.687
rh ant. cingulate gyrus WM	1.012	0.613	0.035*	0.901	0.652	0.256
lh ant. cingulate gyrus WM	0.955	0.727	0.237	1.027	0.837	0.340
rh post. cingulate gyrus WM	0.740	0.643	0.634	0.713	0.690	0.918
lh post. cingulate gyrus WM	0.769	0.651	0.563	0.860	0.662	0.337
rh frontal WM	1.067	1.021	0.862	1.016	0.908	0.597
lh frontal WM	0.986	0.954	0.895	1.017	0.940	0.696
rh parietal WM	0.949	0.687	0.172	0.923	0.707	0.333
lh parietal WM	0.879	0.704	0.367	0.937	0.734	0.337
rh thalamus low T2 intensity	0.371	0.094	0.151	0.514	0.157	0.085
lh thalamus low T2 intensity	0.501	0.323	0.361	0.316	0.202	0.577
rh claustrum	infinity	infinity	-	1.237	1.192	0.815
lh claustrum	1.237	1.192	0.862	infinity	infinity	-
